# Improving Breast Cancer Outcomes Through Quality Care: Call to Action for the Implementation of the Breast Cancer Care Quality Index (BCCQI)

**DOI:** 10.3390/ijerph23020207

**Published:** 2026-02-06

**Authors:** Maira Caleffi, Mary Ajango, Aydah M. Al-Awadhi, Ricki Fairley, Andrea B. Feigl, Ana Rita González, Victoria Harmer, Naveena Nekkalapudi, Toyin Saraki, Victoria Wolodzko Smart, Araceli Fernandez-Cerdeño, João Victor Rocha, Ilaria Lucibello, Namita Srivastava

**Affiliations:** 1 Hospital Moinhos de Vento, Porto Alegre 90560-032, RS, Brazil; maira.caleffi@hmv.org.br; 2 Young Survival Coalition (YSC), New York, NY 10174, USA; majango@youngsurvival.org; 3 Division of Hematology and Medical Oncology, Sheikh Shakhbout Medical City, Abu Dhabi P.O. Box 11001, United Arab Emirates; aydah.alawadhi@gmail.com; 4 Department of Internal Medicine, College of Medicine and Health Sciences, United Arab Emirates University, Al Ain P.O. Box 17666, United Arab Emirates; 5 TOUCH, The Black Breast Cancer Alliance, Annapolis, MD 21403, USA; ricki@touchbbca.org; 6 Health Finance Institute, 3100 Clarendon Blvd, Ste 200, Arlington, VA 22201, USA; andrea@healthfinanceinstitute.org; 7 Policy Wisdom LLC, Quebradillas 00678-2705, Puerto Rico; argonzalez@policywisdom.com (A.R.G.); afernandez@policywisdom.com (A.F.-C.); jrocha@policywisdom.com (J.V.R.); nsrivastava@policywisdom.com (N.S.); 8 Imperial College Healthcare NHS Trust, London W2 1NY, UK; victoria.harmer@nhs.net; 9 Health Consumer Advocate (Independent), Melbourne, VIC 3104, Australia; navvy756@hotmail.com; 10 The Wellbeing Foundation Africa, Lagos 106104, Nigeria; toyin.saraki@wbfafrica.org; 11 Susan G. Komen Breast Cancer Foundation, Dallas, TX 75380, USA; vsmart@komen.org

**Keywords:** breast cancer, Care Quality Index, BCCQI, early detection, timely diagnosis, healthcare systems strengthening, health equity, patient-centered care, quality of care, Call to Action, cancer care policy

## Abstract

**Highlights:**

**Public health relevance—How does this work relate to a public health issue?**

- Breast cancer is the most common cancer among women globally, with increasing incidence, substantial mortality, and economic burden.
- Breast cancer has wide-ranging impacts, affecting individuals’ mental and physical health, disrupting household stability, productivity, equity, and social well-being, and placing strain on healthcare systems.

**Public health significance—Why is this work of significance to public health?**

- Most countries are not on track to achieve global targets for reducing breast cancer mortality, underscoring major gaps in care quality and access, and highlighting the urgency of addressing these issues.
- This paper supports the implementation of the Breast Cancer Care Quality Index, a practical framework to address persistent global inequalities through the assessment and improvement of breast cancer care quality.

**Public health implications—What are the key implications or messages for practitioners, policy makers and/or researchers in public health?**

- Countries can leverage this Call to Action to prioritize context-appropriate interventions through a structured, tiered self-assessment approach applicable across the breast cancer care continuum, including early detection, timely diagnosis, comprehensive management, and broader healthcare system components.
- By aligning actions around essential care elements, the Call to Action helps stakeholders identify priorities, foster coordination, and develop actionable roadmaps that translate commitments into measurable improvements and high-quality care for all women.

**Abstract:**

Breast cancer is the most common cancer among women worldwide and a leading cause of mortality. Stark differences in outcomes across income levels, regions, population groups, and healthcare systems reflect deep inequities in access to early detection, diagnosis, and treatment. Due to remarkable scientific advances and many global initiatives, breast cancer is often perceived as a “finished agenda”. This Call to Action, led and endorsed by a multidisciplinary panel of international experts in breast cancer care, policy, and healthcare systems, provides a structured approach to guide countries in improving breast cancer care through the Breast Cancer Care Quality Index (BCCQI), a unified, expert-endorsed tool that translates broad guidance into practical metrics. The Call to Action outlines a framework for country profiling across the BCCQI dimensions: early detection, timely diagnosis, comprehensive management, and strong and resilient healthcare systems. Applying a structured self-assessment matrix linked to tiered recommendations, the Call to Action supports country performance assessment and the development of context-sensitive roadmaps for concrete interventions. By linking assessment to actionable guidance, the Call to Action underscores the urgency of coordinated national efforts and international support to close existing gaps and accelerate progress toward high-quality breast cancer care for all patients.

## 1. Breast Cancer: An Unfinished Agenda

### 1.1. Background

Breast cancer is the most common cancer among women globally and a major cause of mortality [1]. In 2022, it led to 2.3 million new diagnoses and 670,000 deaths [2]. By 2040, breast cancer cases are projected to exceed 3.19 million annually, with deaths rising to 1.04 million [3]. Key global and regional epidemiological trends in breast cancer reveal significant disparities in incidence, prevalence, and mortality. Incidence rates are highest in North America, Oceania, and Europe, while mortality is highest in Africa. Table 1 presents breast cancer incidence, mortality, and prevalence, per region, with data from 2022 [4].

Over the last few decades, significant technological and clinical advances have changed the breast cancer care pathway and improved the prognosis for many patients [5]. In addition, international health organizations have developed evidence-based and resource-adapted programs and interventions to support countries, healthcare systems, and institutions in improving breast cancer outcomes worldwide.

The Breast Health Global Initiative, the Breast Cancer Initiative 2.5, and the recent Global Breast Cancer Initiative (GBCI)—launched in 2021 by the World Health Organization (WHO), in collaboration with the International Atomic Energy Agency and the International Agency for Research in Cancer [6]—are only a few among them. This collective effort has contributed to the perception that breast cancer is a “finished agenda”, shifting attention toward other priorities.

Yet the 2024 Lancet Breast Cancer Commission highlighted ongoing inequities [5], and recent data shows that among 185 countries, only seven of the most developed, predominantly high-income countries (HICs), are meeting—and six are close to meeting—the GBCI goal to reduce breast cancer mortality by 2.5% annually [1,7].

Around 99% of breast cancer cases occur in women [2]. Outcomes vary widely across income levels, population groups, regions, and healthcare systems [8]. Some of these variations reflect intrinsic factors—such as population structure, tumor biology, and genomics [9,10]. However, most differences arise from system-level inequities, including inconsistent access to innovations and variations in the quality of early detection, diagnosis, and treatment services [11,12]. These gaps are often rooted in broader gender and socioeconomic inequities [5,8,11,12].

Women in low- and middle-income countries (LMICs) are less likely to be diagnosed and more likely to die from the disease [2]. Data show that women in developing countries experience 17% higher mortality compared to those in advanced economies [8]. This is critical as many of these countries face stagnant or even rising death rates [5,13,14].

These disparities are even more pronounced among women in humanitarian crises, including displaced and refugee populations [15].

Breast cancer therefore serves as a sentinel condition for gender-responsive, equitable care, revealing systemic gaps that disproportionately impact vulnerable women across all levels of development.

This highlights that closing the gaps in breast cancer quality of care is a high priority, not only for reducing preventable mortality but also for addressing the broader structural inequalities that drive these disparities [16]. Beyond the equity imperative, there is also a compelling economic case for action with proven economic returns on investment from strengthening breast cancer care, including improved productivity, household stability, and reduced catastrophic expenditure [16,17].

Without decisive action, the growing burden of breast cancer will deepen existing inequalities [18,19] and strain limited resources, making reduction in breast cancer mortality increasingly difficult. To this end, bold policy action and sustained commitment are essential to improve the quality of breast cancer care worldwide [17], highlighting the urgency to act now. As highlighted in Table 2, this also closely aligns with the 2030 Sustainable Development Agenda [20], making renewed attention to breast cancer a pivotal priority.

The aim of this Call to Action, co-authored and endorsed by a multidisciplinary panel of healthcare providers, policy and public health experts, patient advocates, and patients, is to provide a structured framework to guide countries in implementing the Breast Cancer Care Quality Index (BCCQI) [21]. The BCCQI is an expert-endorsed, structured tool developed through comprehensive literature review and a multistep process of expert consultation and validation. It aims to drive actionable and systematic healthcare transformation, closing gaps in breast cancer care and accelerating measurable improvements in outcomes. This underscores the need for bold policy action and sustained, long-term commitment.

### 1.2. Challenges in Breast Cancer Care

**Figure 1:**
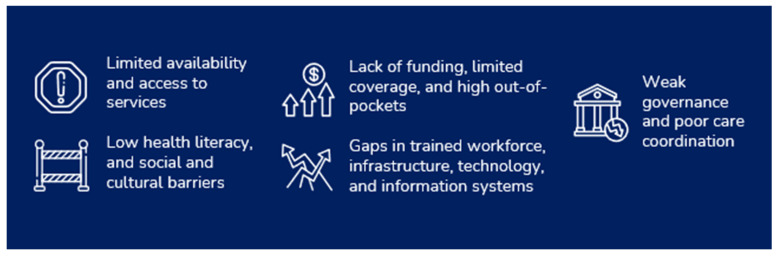
Overview of key challenges in breast cancer care.

Numerous challenges affect breast cancer care, becoming significant determinants of the discussed disparities (see Figure 1 below).

Early detection is critical, as identifying breast cancer at an earlier stage greatly improves survival outcomes and significantly reduces cost through more effective treatment options. However, behavioral and psychosocial factors, such as stigma, fear, gender inequity, low health literacy, and misconceptions about cancer hinder early detection [1,11,22,23,24]. These barriers are further exacerbated by critical gaps in primary healthcare, which constitutes the backbone of integrated care and enables coordination and patient navigation from early detection to survivorship and follow-up. Documented systemic challenges that hamper the quality of primary healthcare services include limited access, difficulty scheduling appointments, and transportation barriers [11]. In addition, patients face limited symptom awareness, a low quality of services provided, and inadequate diagnostic tools, which can delay timely detection after symptom onset [1,22,25,26,27,28]. In rural and underserved contexts, community health workers could play an important role in addressing barriers to early breast cancer detection [29].

Barriers to timely breast cancer diagnosis—whether individual, disease-related, or systemic—can delay completion of appropriate diagnosis and staging, adversely affecting patient prognosis. Diagnostic delays after referral can result from low health literacy, limited disease awareness, and socio-economic factors [11,30,31]. Financial barriers, limited support, and travel challenges related to low income, unemployment, marital status, and distance from healthcare facilities can further extend diagnostic timelines [11,30,31,32,33].

Treatment is central to improving survival and quality of life, with timely initiation and high-quality care substantially reducing mortality and recurrence risk. Multiple patient-related, financial, and systemic barriers delay treatment initiation, impact access to optimal and comprehensive care, and hinder patient compliance with treatment for breast cancer, especially among vulnerable populations [11,34,35,36]. These challenges contribute to poorer outcomes and widen the equity gap in breast cancer care [37]. Misconceptions, fear, and high out-of-pocket costs contribute to inequities both within and across countries. In addition, unmet needs in supportive care, such as psychological support, pain management, and palliative care can persist in both LMICs and HICs. These gaps, often worsened by low awareness, misconceptions, and financial barriers, can lead to treatment interruptions or abandonment [1,35,38]. The limited use of patient-reported outcomes and patient-reported experience measures (PROMs and PREMs) further hinders understanding of breast cancer impact on health-related quality of life of patients [39].

Strong and resilient healthcare systems are essential for delivering high-quality breast cancer care, as they influence every stage of the patient’s journey. Weak governance, limited financing, and service delivery challenges hinder access to quality breast cancer care [23,36,40]. In many countries, the absence of national cancer plans, sustainable funding, and essential diagnostics, medicines, and equipment contributes to late-stage diagnoses and suboptimal treatment outcomes [1,40,41,42]. Workforce shortages, poor care coordination, geographical barriers, long waiting times, and inconsistent adherence to clinical practice guidelines further compromise care quality [11,30,36,43,44,45,46]. Weak health information systems and limited healthcare professional skills additionally constrain the healthcare system’s capacity to deliver timely, equitable, and high-quality care [23,36,47].

## 2. Need for Action

Despite decades of progress, breast cancer care remains marked by persistent gaps that limit survival gains and widen inequities. While current initiatives have contributed valuable advances, they have too often failed to translate scientific and clinical progress into tangible improvements in outcomes for patients worldwide. This underscores an urgent need for approaches that effectively drive system-wide change.

The Breast Cancer Care Quality Index (BCCQI) offers such an approach [21]. As a catalyst for progress, the BCCQI helps reframe breast cancer as an unfinished agenda and provides a unifying framework that bridges existing initiatives by translating broad guidance into practical, actionable measures. It empowers policymakers, clinicians, and advocates to align around a common standard and accelerate change by enabling countries to design context-sensitive roadmaps and implement tailored actions that foster stepwise improvements across the full continuum of breast cancer care. Furthermore, recognizing the widespread gender inequities that affect healthcare quality, the BCCQI was built to provide solutions that promote more equitable governance.

A global Call to Action to implement the BCCQI is now imperative. Embedding the BCCQI into breast cancer strategies is essential to ensure commitments translate into results. Designed to accelerate progress, the BCCQI can help countries prioritize policies and interventions that drive equitable, effective, and sustainable improvements in breast cancer care worldwide (Box 1).

Box 1 **Key Breast Cancer Care Quality Index (BCCQI) messages.**

**The BCCQI matters now**

Breast cancer remains an unfinished agenda, with widening inequities and slow progress toward global mortality-reduction targets. The BCCQI provides a unified, actionable framework to turn commitments into measurable improvements.

**What adoption will deliver in 3–5 years**

Countries can accelerate earlier diagnosis, shorten diagnostic delays, improve treatment initiation and completion, and strengthen quality and accountability systems aligned with Universal Health Coverage and SDG goals through the development of tailored breast cancer roadmaps.

**Immediate commitments for ministers**

Endorse national BCCQI adoption, mandate a time-bound implementation roadmap, secure sustainable financing, and embed BCCQI indicators into national monitoring and cancer registry systems to drive rapid, accountable progress.

## 3. Introducing the Breast Cancer Care Quality Index (BCCQI)

The Breast Cancer Care Quality Index (BCCQI) is structured into dimensions, goals, targets, and indicators, mirroring the structure of key outcome-driven health-related frameworks established by international organizations and their collaborators. The BCCQI has four dimensions: early detection, timely diagnosis, comprehensive management, and strong and resilient healthcare systems. Each dimension has an associated goal, which outlines broad objectives, defining the desired achievements within each dimension. Targets specify measurable outcomes with defined timeframes, articulated through clear statements or quantitative benchmarks. Achieving these targets is critical to fulfilling the overarching goals. Indicators are the metrics used to monitor progress, assess gaps, and measure advancement toward targets. The BCCQI was primarily developed to address the challenges presented in Figure 1, also being guided by five essential domains aligned with global health priorities: health equity, patient centricity, universal access to healthcare, healthcare quality, and effective treatment. Table 3 summarizes the BCCQI targets and indicators by dimension, including the domains each indicator aims to enhance [21].

The BCCQI further supports and strengthens action under the GBCI and other relevant initiatives, enhancing their impact [21]. Recognizing that countries differ in context, investment capacity, and priorities, the BCCQI functions as a flexible and iterative tool that enables adaptation to national needs and resources. This approach facilitates the development of context-sensitive roadmaps and allows for phased national adoption through pathways tailored to each country’s healthcare system and capacity. To achieve this, it is essential to assess the varying levels of breast cancer progress across countries to identify gaps and opportunities. The following sections support the identification of areas requiring attention, prioritization of actions, and the development of country-specific roadmaps reflecting recommendations to advance breast cancer care.

## 4. Country Profiling Using the Breast Cancer Care Quality Index (BCCQI)

To support countries in applying the Breast Cancer Care Quality Index (BCCQI), a four-tier framework was introduced that characterizes typical stages of progress in breast cancer care across the four BCCQI dimensions. Each stage reflects the possible level of advancement across policy, services, and outcomes for each dimension. These reference profiles are intended to support national stakeholders in assessing the current state of breast cancer care. By comparing their policy, service, and outcome status against those described for each tier, countries can identify the group that most closely represents their situation in each dimension and recognize areas where improvements are needed.

To provide relevant guidance at the country level, a preliminary analysis was conducted to demonstrate how country-level assessments using the BCCQI could be operationalized in practice.

Given the absence of comprehensive national data required for direct BCCQI application, this analysis relied on globally available and methodologically consistent datasets. While data gaps remain a major barrier to progress in breast cancer care, the analysis recognizes the need to begin assessing the current situation, identify specific gaps, and build strategies to address them.

Supplementary Material S1 presents this exploratory 100-country application, detailing the indicator set (Table S1), scoring criteria (Table S2), and regional country rankings (Tables S3–S6). This exercise illustrates how structured frameworks like the BCCQI can guide the development of tailored recommendations based on countries’ progress toward common indicators.

The analysis demonstrates that even without consistent data addressing all BCCQI indicators, countries can advance framework implementation. This underscores the need for a global self-assessment questionnaire to enable coordinated baseline and progress reporting.

Recognizing that imperfect data systems must never delay action, these preliminary tools can help countries address data gaps while generating evidence to validate the BCCQI across diverse contexts. This process provides insights into a country’s current state, determines its level of progress across the dimensions and their specific elements, and highlights critical gaps requiring attention.

Based on these findings, the analysis seeks to provide the following: (i.) an initial structured matrix for self-assessment designed to support countries toward developing a formal, context-sensitive roadmap and (ii.) practical guidance for them to assess their current level of progress in each dimension of the BCCQI.

Progress towards improved breast cancer care might vary across dimensions, targets, and indicators. Countries may not fully align with a single profile but may instead exhibit elements from different profiles. In such cases, countries should identify the closest overall grouping and draw on relevant recommendations appropriate to the progress level of each aspect. This reflects the reality that countries may align with elements of different tiers simultaneously, as movement between tiers does not occur in a linear manner.

The framework is therefore designed to accommodate such heterogeneity, allowing countries to position themselves at different levels for early detection, timely diagnosis, comprehensive management, and healthcare system strengthening and resilience, in alignment with their actual situation. This approach enables countries to select context-specific priorities and recommendations based on their unique profiles, supporting national stakeholders and decision makers in identifying realistic advancement roadmaps and optimizing resource allocation—a capability not previously available in global breast cancer policy assessment. Establishing this baseline enables stakeholders to leverage the group-specific recommendations presented in Section 6 to select critical actions to prioritize and include in their final BCCQI country-specific roadmap.

Table 4, Table 5, Table 6 and Table 7 summarize the features and characteristics of countries achieving *low, modest, moderate,* and *outstanding* levels of performance for each dimension.

Table 4 specifically focuses on early breast cancer detection. Each level is defined by the presence and scope of national policies, awareness and education programs, the existence of an operational definition of “women at elevated risk”, the use of risk-stratified screening strategies, referral pathways, and the proportion of cancers detected at early stages (I–II). Country-level characteristics illustrate the context-specific features, such as equity of access and monitoring practices. The table provides a structured framework for assessing progress and identifying gaps in early detection capacity and implementation.

Following the assessment of early detection capacity, Table 5 outlines the typical profiles for the dimension of timely breast cancer diagnosis. It categorizes countries based on the presence and implementation of suitable policies for breast cancer diagnosis, the proportion of patients receiving a complete diagnosis and staging within two months from initial presentation due to a suspicious finding, and the availability and equity of quality diagnostic services. The table highlights differences in policy and outcomes, providing a framework to evaluate how effectively healthcare systems translate early detection into timely and complete diagnosis.

Building on the progression from early detection to timely diagnosis, Table 6 shifts the focus to the dimension of comprehensive breast cancer management, which comprises multidisciplinary care, supportive services such as pain management, physiotherapy, lymphedema management, psycho-oncology, and oncofertility, as well as survivorship plans. The four levels of achievement in this dimension are based on the scope and enforcement of national policies guaranteeing comprehensive breast cancer management, timeliness and equity of treatment initiation, access to subtype-specific therapies, integration of supportive care, and incorporation of survivorship planning. The profiles capture, among other things, differences in treatment completion rates, availability of multidisciplinary teams, use of patient-reported outcomes, and continuity of care, offering a structured lens to assess how healthcare systems deliver and sustain high-quality breast cancer care beyond diagnosis.

Concluding the sequence of dimensions, Table 7 presents typical profiles of progress for strong and resilient healthcare systems that are well suited to sustain and scale breast cancer control efforts. It classifies countries according to their maturity of funding mechanisms, workforce capacity, infrastructure, data systems, guideline implementation, care coordination, and patient engagement channels. The profiles emphasize how system-level strength underpins the effectiveness of early detection, timely diagnosis, and comprehensive management by ensuring consistent quality, equity, and sustainability across the entire care continuum.

Taken together, these four sets of profiles offer countries a practical assessment matrix to evaluate their current level of achievement across the four BCCQI dimensions. By identifying the profile that most closely reflects their current situation, policymakers and multisectoral stakeholders can pinpoint critical gaps and opportunities for progress. The following section builds on this assessment, presenting tailored recommendations for each level of achievement in every dimension. These recommendations aim to help align priorities and foster collaboration among different stakeholders at the country level and beyond.

## 5. Strategic Recommendations: Scaling up the Breast Cancer Care Quality Index (BCCQI)

Building on the country-level analyses, a structured set of recommendations is proposed across each dimension of breast cancer control to support the translation of evidence into policy and practice. Collectively, these recommendations aim to guide countries in addressing persistent system gaps, improving quality of care, and reducing inequities in outcomes. Specifically, they are designed to accomplish the following:

- Meet the Breast Cancer Care Quality Index (BCCQI) targets;
- Improve performance on key indicators;
- Align stakeholders at both national and international levels around common priorities.

Each recommendation outlines activities to foster advocacy and coordinated action by policymakers and multisectoral stakeholders, accelerating improvements across the breast cancer care continuum. By favoring alignment on critical actions to undertake, these recommendations can build consensus, generate urgency, and create a strong foundation for mobilizing support from the international community. Key stakeholders responsible for implementation are outlined in Table 8.

Policymakers provide the formal authority, coordination capacity, and resource allocation needed to integrate improvements in breast cancer care into national strategies and regulatory frameworks.

Multisectoral stakeholders contribute essential expertise, operational capabilities, and community reach for effective implementation. Within this group, medical and scientific societies, knowledge-based and academic institutions, professional associations, and the private sector support evidence generation, guideline development, training, and innovation. Civil society, survivors, and women’s organizations provide critical insights and networks that promote awareness, adherence, and accountability, ensuring that patient, survivor and caregiver voices shape national policies, plans, and review cycles.

Finally, the international community strengthens these efforts through global guidance, technical support, and financial and convening capacity. It also legitimizes country efforts by highlighting alignment with global priorities and agendas, helping advance improvements in breast cancer care.

The goal is for countries to select a set of recommendations for each dimension based on their respective level of progress in consideration of their local needs, opportunities, and challenges, ultimately developing true context-sensitive roadmaps, tailored to their specific contexts, resources, and healthcare system realities.

For each recommendation, Supplementary Material S2 provides the expanded stakeholder typology and a crosswalk identifying which actors should develop, implement, provide technical expertise, and advocate for each tiered recommendation across Dimensions A–D (Tables S7–S23). This clarifies roles and responsibilities to support coordinated implementation. By doing so, Supplementary Material S2 helps ensure that responsibilities are clearly defined and collaboration is optimized to drive meaningful and sustainable progress.

Table 9 provides a tiered menu of recommendations for early breast cancer detection, aligned to each level of progress as described in Table 4. The proposed actions are linked to the specific targets and indicators of Dimension A: early breast cancer detection and can serve as a practical guide for selecting interventions, strengthening multisectoral collaboration, and accelerating measurable progress. Each recommendation can be further adapted to the country context, if needed.

Building on early detection efforts, the recommendations in Table 10 address the critical needs under Dimension B: timely breast cancer diagnosis. While identifying suspicious findings is essential, it does not translate into improved outcomes unless it is promptly followed by a complete and accurate diagnostic assessment. Table 10 provides guidance for countries across all levels of system maturity (as outlined in Table 5) to ensure that every woman with a suspicious finding receives a full diagnostic work-up within the recommended 60-day timeframe.

Following timely diagnosis, it is essential to guarantee access to high-quality, comprehensive breast cancer care across the whole treatment pathway and beyond. Table 11 presents recommendations related to Dimension C: comprehensive breast cancer management, which aims to support countries at different levels of achievement—as outlined in Table 6—in implementing and scaling multidisciplinary treatment services. This dimension of the BCCQI covers different types of services which are critical for comprehensive care: from multidisciplinary treatment to supportive services and survivorship care, all critical components for the delivery of high-quality breast cancer care. The full range of essential services is fully articulated in the BCCQI framework, but it can be further expanded through the inclusion of other evidence-based services, once countries have achieved optimal delivery of the critical services pinpointed by the BCCQI co-authors in their work [21]. Patient-centeredness, which is also emphasized by the BCCQI, is also fully acknowledged in this dimension with the inclusion of specific focus on patient-reported outcomes and experience measures (PROMs and PREMs).

While advancing these types of treatments can seem far-fetching for resource-constraint settings, the relevant activities proposed in Table 11 indicates that, if a country identifies this as a priority area, context-appropriate solutions exist that can be adapted and replicated. For example, PROMs and PREMs can be introduced through short, paper-based surveys administered at key clinical touchpoints.

This has been demonstrated in Kenya where 66% of cancer patients self-administered the European Organisation for Research and Treatment of Cancer Quality of Life Questionnaire Core 30 with a mean completion time of 13 min. This is a questionnaire measures cancer patients’ health-related quality of life using 30 items covering five functioning domains (physical, role, cognitive, emotional, social), key symptoms (fatigue, pain, nausea/vomiting), global health status/quality of life, and six single items (dyspnea, appetite loss, sleep disturbance, constipation, diarrhea, and financial impact) [48].

Similarly, in Ethiopia, an oncology center collected patient-experience data using an interviewer-administered European Organisation for Research and Treatment of Cancer satisfaction with cancer care core questionnaire. This tool measures oncology outpatients’ experience and satisfaction with care using 33 items covering provider performance (doctors’ technical skills, information exchange, affective behavior), satisfaction with nurses and radiotherapy technologists, care coordination, interactions with hospital staff, and hospital organization and environment [49].

Overall, the actions outlined in Table 11 aim to ensure that patients not only start but also complete appropriate therapy, aligned with international standards and tailored to their specific cancer subtype.

Effective early detection, diagnosis, and treatment all depend on the existence of strong, resilient healthcare systems. Table 12 presents recommendations related to Dimension D: strong and resilient healthcare systems, aiming to strengthen the backbone of breast cancer care—starting from each country’s specific level of progress, as described in Table 7—covering infrastructure, workforce, financing, and governance. These actions involve system-wide investments in healthcare system capacity, including cancer registries, patient navigation, clinical guidelines, and institutionalized patient engagement, among others. They ensure sustainability, equity, and quality of care delivery across all levels of the system and the continuum of breast cancer care.

Together, the recommendations outlined in Table 9, Table 10, Table 11 and Table 12 offer a tool to help build a comprehensive roadmap to accelerate progress across all dimensions of breast cancer care, from early detection through treatment completion to system-level resilience. Countries can and should begin implementing the BCCQI even with partial data. Data gaps and inadequate data system must never delay action; instead, data strengthening and implementation can progress in parallel. For example, resource-constrained countries can leverage existing cancer registry data, even when incomplete, as well as alternative data sources that may serve as proxies to initiate assessments. Where feasible, self-assessment questionnaires completed by country officials or national experts may also help address data gaps. Finally, the BCCQI can support national data-strengthening efforts by serving both as an assessment framework and as a catalyst for resource mobilization to improve data availability and quality over time.

Box 2 illustrates this process by presenting a hypothetical country example, showing how the self-assessment process, as presented in Table 4, Table 5, Table 6 and Table 7, informs the subsequent development of recommendations, as presented in Table 9, Table 10, Table 11 and Table 12.

Box 2 **Applying the Call to Action self-assessment matrix and tiered recommendations to develop a baseline and roadmap for the BCCQI early detection dimension (hypothetical “Country X”).**

**Country X: Description**

Country X has recently adopted a national policy for the early detection of breast cancer; however, while the policy establishes a general population screening campaign, it does not define women at elevated risk of breast cancer nor establish targeted screening approaches for these populations. Awareness and education programs have been implemented by international non-governmental organizations with support from international financing and technical partners such as the World Bank and the WHO and have been incorporated into the recently adopted national policy. As a result of these initiatives, awareness among the primary and community-based health workforce is generally adequate. Nevertheless, considerable gaps persist in the systematic administration of individual risk assessment, as specific tools and guidance for this purpose are lacking. Moreover, the timely referral of women with suspicious findings through appropriate care pathways remains inconsistent.

The country’s health data system remains very weak, with only fragmented data collected. Despite the existence of primary health or community-level services for early identification and referral, access remains limited for some of the most vulnerable populations, including members of a large native ethnic group at high risk of breast cancer who live in a remote area of the country. As a result, estimates indicate that more than 75% of invasive breast cancers are diagnosed at stage III or IV according to TNM anatomical or pathological staging.

**Country X: Profile**

Based on the typical policy progress profiles presented in Table 4 for early detection of breast cancer, Country X meets the criteria for countries with a *Modest* level of achievement. However, with respect to the proportion of invasive breast cancers diagnosed at stage I or II according to TNM anatomical or pathological staging, the country meets the criteria for a *Low* level of achievement.

**Criteria for Modest Level of Achievement met by Country X:**

- Drafted or recently adopted national policy or framework, outlining basic measures for early detection.
- Awareness and education programs exist but are fragmented or pilot-based, not integrated into national health frameworks.
- Some primary health or community-level services attempt early identification and referral, but pathways have not been formally established.
- No definition of women at elevated risk and targeted screening limited to certain jurisdictions, or dependent on external funding.

**Criteria for Low Level of Achievement met by Country X:**

- <25% of invasive breast cancers diagnosed at stage I or II according to TNM anatomical or pathological staging or no data for this indicator.

**Country X: Potential activities for a short–medium-term roadmap**

Based on the recommendations for early breast cancer detection presented in Table 9 and the specific features outlined in the country description, Country X could consider the following activities, which are applicable to countries with *Low* or *Modest* levels of achievement, as outlined below.

**Recommendations for Low Level of Achievement applicable to Country X:**

- **Develop risk-assessment tools:** With the support of national or international stakeholders, develop context-specific tools for individual risk assessment to be administered to women during GP visits.
- **Establish referral pathways:** Pilot basic referral pathways for suspicious findings in high-population areas as a model for future scale-up to reach rural or marginalized jurisdictions.

**Recommendations for Modest Level of Achievement applicable to Country X:**

- **Promote implementation of breast cancer policy:** Advocate for stepwise institutionalization of measures from the proposed or recently adopted breast cancer detection plan.
- **Develop a definition of women at elevated risk of breast cancer:** Develop an evidence-based definition of women at elevated risk of breast cancer to delineate the target population for the national early detection strategy and prioritize higher-frequency screening for this group.
- **Develop a definition of women at elevated risk of breast cancer:** Develop an evidence-based definition of women at elevated risk of breast cancer to delineate the target population for the national early detection strategy and prioritize higher-frequency screening for this group.
- **Standardize risk-assessment in primary healthcare:** Develop context-specific tools for individual risk-assessment to be administered to women during GP visits and establish national breast cancer risk-assessment protocols to be rolled out to GPs at the national level.
- **Expand public and workforce awareness:** Strengthen health promotion efforts, such as early detection awareness campaigns for the public, and scale education programs for healthcare workers by integrating breast cancer modules into existing national training platforms.

It is important to recognize that besides data constraints, other barriers, often not strictly related to breast cancer, may limit adoption of the context-sensitive roadmaps and the tailored recommendations [21]. These include limited public awareness and advocacy, cultural stigmas, logistical bottlenecks, workforce and infrastructure gaps, and financial limitations that can hinder political will. Geopolitical instability, competing priorities, and external pressures such as economic shocks, further risk deprioritizing the implementation of the framework outlined in this Call to Action and the adoption of the BCCQI.

Yet, by helping adapt the efforts to country’s context and actual level of achievement, the framework supports action and helps overcome also these barriers, facilitating the identification of realistic, tailored pathways for sustained, incremental improvements towards reduced disparities, improved outcomes to ensure that every woman can access timely, high-quality breast cancer services, irrespective of where they live.

## 6. Conclusions

Persistent gaps in breast cancer care continue to limit improvements in outcomes and wide inequities, indicating that current efforts have not fully translated progress into consistent, system-wide benefit, which highlights the need for bold action.

Breast cancer care remains an unfinished agenda, with disparities in access, quality, and outcomes threatening progress worldwide. By supporting the practical application of the Breast Cancer Care Quality Index (BCCQI), this Call to Action establishes a practical, structured approach for countries to move from knowledge to action.

Through the framework of Table 4, Table 5, Table 6 and Table 7, countries can assess their current level of progress across early detection, diagnosis, comprehensive care, and healthcare system resilience. The tiered recommendations in Table 9, Table 10, Table 11 and Table 12 then provide actionable guidance for national stakeholders to address gaps, prioritize interventions, and build a context-sensitive implementation roadmap tailored to their context and healthcare system realities.

For countries to be ready for the rising burden of breast cancer, urgent action is required: they must adopt the BCCQI as a strategic tool to identify gaps, translate commitments into measurable improvements. Multisectoral collaboration and international support, through varied forms of cooperation, such as pooled global technical assistance, shared self-assessment tools, cross-country learning platforms, or mentorship networks, could be leveraged to support these efforts. Through this approach, progress in breast cancer care can be accelerated, disparities reduced, and sustainable, high-quality care ensured. By bridging the gap between global targets and national action, healthcare system transformation becomes possible, and this Call to Action provides a structured framework to make it a reality—ensuring that no woman is left behind.

## Figures and Tables

**Table 1:** Breast cancer incidence, mortality, and prevalence, per region, 2022.

Region	Incidence Absolute Value	Incidence Age Standardized Rate Per 100,000	Mortality Absolute Value	Mortality Age Standardized Rate Per 100,000	5-Year Prevalence Absolute Value
Global	2,296,840	46.8	666,103	12.7	8,178,393
Asia	985,817	34.3	315,309	10.5	3,197,043
Europe	557,532	75.6	144,439	14.6	2,296,495
North America	306,307	95.1	91,252	12.3	1,332,343
Latin America and the Caribbean	220,124	52.0	59,876	13.2	725,017
Africa	198,553	40.5	49,744	19.2	507,659
Oceania	28,507	91.5	5483	15.4	119,836

**Table 2:** Breast cancer and alignment with the 2030 Sustainable Development Agenda.

Sustainable Development Goals (SDGs) and Targets	Goal/Target
SDG 3.4	By 2030, reduce by one third premature mortality from non-communicable diseases through prevention and treatment and promote mental health and well-being
SDG 3.8	Achieve universal health coverage, including financial risk protection, access to quality essential health-care services and access to safe, effective, quality and affordable essential medicines and vaccines for all
SDG 5	Achieve gender equality and empower all women and girls
SDG 10	Reduce inequality within and among countries

**Table 3:** Overview of the Breast Cancer Care Quality Index (BCCQI).

Dimension Identification, Dimension, and Goals	Target/ Indicator Identification	Target/Indicator Focus
**A: Early Breast Cancer Detection** *Promote early detection of breast cancer*	A.1	**Existence of national programs, policies, or frameworks for early breast cancer detection.**
	A.1.1	Existence of national program, policy, or framework.
	A.1.2	Inclusion of breast cancer awareness and education programs for the public and healthcare workers in national health-related frameworks.
	A.2	**Availability of and access to breast cancer early detection programs and services.**
	A.2.1	Execution of breast cancer early detection programs and services.
	A.2.2	QUANTITATIVE INDICATOR: Proportion of women at elevated risk of breast cancer screened at least once every two years.
	A.3	**Ensure that at least 60% of invasive breast cancers are diagnosed at stage I or II.**
	A.3.1	QUANTITATIVE INDICATOR: Proportion of invasive cancers diagnosed at stage I or II according to (Tumor, Nodes, and Metastasis) TNM anatomic and/or pathological staging.
**B: Timely Breast Cancer Diagnosis** *Ensure timely access to appropriate breast cancer diagnosis*	B.1	**Access to diagnostic services.**
	B.1.1	Existence of a national policy or framework for breast cancer diagnostic services.
	B.2	**Ensure diagnosis completion within two months from first access/presentation due to a suspicious finding.**
	B.2.1	QUANTITATIVE INDICATOR: Proportion of patients with complete diagnosis and appropriate staging within two months from first access/presentation due to a suspicious finding.
**C: Comprehensive Breast Cancer Management** *Guarantee timely access to comprehensive breast cancer treatment and care for all patients at all stages*	C.1	**Access to breast cancer care and management.**
	C.1.1	Existence of a national policy or framework for access to breast cancer care.
	C.1.2	QUANTITATIVE INDICATOR: Proportion of patients with timely treatment initiation.
	C.1.3	QUANTITATIVE INDICATOR: Proportion of triple-negative breast cancer, HER2+, and (Hormone Receptor positive / HER2 negative) HR+/HER- early breast cancer patients who receive adequate treatment.
	C.1.4	QUANTITATIVE INDICATOR: Proportion of patients with hormone receptor-positive invasive breast cancer who receive adequate treatment.
	C.1.5	QUANTITATIVE INDICATOR: Proportion of breast cancer patients that receive adequate supportive services.
	C.1.6	Incorporation of patient perspective in service quality assessment protocols for breast cancer.
	C.1.7	Existence of a national program, policy, or framework for survivorship care plan implementation.
	C.2	**Ensure treatment completion for more than 80% of breast cancer patients.**
	C.2.1	QUANTITATIVE INDICATOR: Proportion of patients who complete their recommended treatment out of the total number of patients treated.
**D: Strong and Resilient Healthcare Systems** *Strengthen overall health system capacity for health promotion, and breast cancer diagnosis, treatment, and care*	D.1	**Strengthen healthcare system’s infrastructure, capability, capacity, knowledge, and resources.**
	D.1.1	Availability of sustainable sources of funding for breast cancer.
	D.1.2	QUANTITATIVE INDICATOR: Number of breast cancer-specialized healthcare professionals per 10,000 cancer patients.
	D.1.3	QUANTITATIVE INDICATOR: Number of specialized hospital units or departments that provide multidisciplinary breast cancer care per 10,000 cancer patients.
	D.2	**Availability of data regarding breast cancer.**
	D.2.1	Existence of population-wide data through national or regional cancer registries.
	D.2.2	QUANTITATIVE INDICATOR: Yearly breast cancer mortality and/or 5-year survival.
	D.3	**Availability and application of clinical practice guidelines and coordination of care mechanisms.**
	D.3.1	Existence of a framework for clinical practice guidelines adoption, dissemination, and adherence.
	D.3.2	Existence of a well-defined service integration/patient navigation mechanism.
	D.3.3	Existence of a framework to ensure patient engagement in healthcare decision-making and health service planning and design.

**Table 4:** Typical policy progress profiles in Dimension A: early breast cancer detection.

**I. Low level of achievement**	**II. Modest level of achievement**
No national policy or framework for early detection exists. No structured awareness or education programs for the public or healthcare workers on early signs and symptoms. No organized programs for early identification and referral of individuals with suspicious findings. No definition of women at elevated risk or targeted screening; any screening is purely opportunistic. <25% of invasive breast cancers diagnosed at stage I or II according to TNM anatomical or pathological staging or no data for this indicator. **Country characteristics:** Early detection depends entirely on individual initiative or private sector. No systematic monitoring of early detection indicators.	Drafted or recently adopted national policy or framework, outlining basic measures for early detection. Awareness and education programs exist but are fragmented or pilot-based, not integrated into national health frameworks. Some primary health or community-level services attempt early identification and referral, but pathways have not been formally established. No definition of women at elevated risk and targeted screening limited to certain jurisdictions, or dependent on external funding. 25–60% of invasive cancers diagnosed at stage I or II. **Country characteristics:** Persistent inequities and limited reach of rural/vulnerable women. Weak linkage between early detection and timely diagnostic confirmation.
**III. Moderate level of achievement**	**IV. Outstanding level of achievement**
National policy or framework is operational and includes standards for awareness, referral, and early detection in primary health or community care facilities. Public and healthcare worker awareness programs are integrated into national health strategies and implemented nationwide. Primary health/community-level facilities routinely identify suspicious findings and refer individuals for diagnostic work-up (with moderate levels of coverage ensured through appropriate programs even among the most vulnerable populations, such as refugees and displaced women). No definition of women at elevated risk of breast cancer, with screening protocols definition based on age-based risk stratification. Pilot stratified screening programs for women at elevated risk underway, but no institutionalized genetic, family history, or personal history risk-stratification in the national screening protocols; frequency aligns with international guidelines. 60–75% of invasive cancers diagnosed at stage I or II. **Country characteristics:** Operational policy and early detection programs. Referral pathways function in most jurisdictions, with a geographic reach covering at least two thirds of the population. Gaps persist in remote areas or among marginalized groups.	Comprehensive national policy fully implemented and monitored, ensuring equitable access to early detection services. Universal, systematic education for public and providers on breast cancer signs, symptoms, and early detection practices. Fully functioning referral pathways ensure prompt identification and diagnostic confirmation across all regions. Adopted operational criteria to define women at elevated risk. Systematic screening of women at elevated risk at least every two years, institutionalized and supported by national protocols (with high levels of coverage ensured through appropriate programs even among the most vulnerable populations, such as refugees and displaced women). ≥75% (ideally ≥90%) of invasive cancers diagnosed at stage I or II. **Country characteristics:** Strong, universal, and well-monitored early detection programs. Minimal disparities across socioeconomic or geographic groups. Strong integration of early detection with timely diagnosis.

**Table 5:** Typical policy progress profiles in Dimension B: timely breast cancer diagnosis.

**I. Low level of achievement**	**II. Modest level of achievement**
No national policy or framework exists regulating access to breast cancer diagnosis and staging. Lack of monitoring for diagnostic delays and quality assessment. Lack of guarantees in support of affordable access to diagnostics; most diagnostic costs paid out-of-pocket. Uneven distribution of diagnostic services, mainly concentrated in urban centers, with inadequate referral systems and minimal support for rural and marginalized populations. Low-quality services with HR/HER2 and pathology often unavailable or unaffordable. Less than 25% of patients receive complete diagnosis and appropriate staging within two months of initial presentation due to a suspicious finding or no data is available on timeliness. **Country characteristics:** Lack of, or ineffective coordination efforts to develop and implement a national policy. Absence of national guidance and standards results in inconsistent quality and uneven delivery of diagnostic services. Inadequate referral systems lead to fragmented diagnostic services, which are mostly available in tertiary centers or the private sector. Significant inequities in access (urban, employed, or high SES vs. rural, marginalized, unemployed, or low SES women). Limited availability of essential diagnostic components (e.g., HR/HER2 testing). Lack of regulated time benchmarks for diagnosis completion and long waiting lists cause significant delays, which may not be monitored.	National policy or framework under development or recently adopted but not fully implemented. Basic diagnostic steps are outlined in policy, with limited standardization and quality oversight. Diagnostic timelines mentioned in policy; some delay-tracking efforts underway. Policies guarantee affordable access to imaging and pathology, but coverage for biomarker, including HR/HER2, or germline testing is inconsistent. Referral pathways exist on paper but are not fully implemented, leading to access challenges in rural areas. Between 25% and 50% of patients achieve complete diagnosis and appropriate staging within two months of initial presentation due to a suspicious finding. **Country characteristics:** National policy emerging or in place, but implementation is weak. Despite improvements in specific jurisdictions or services, diagnostic access and quality remain uneven. Coverage expansion efforts have begun but still exclude critical diagnostic tests. Service fragmentation persists with inconsistent referral and follow-up systems and significant inequities. Incipient systems for monitoring timelines are not yet reflected in performance improvements.
**III. Moderate level of achievement**	**IV. Outstanding level of achievement**
National policy or framework is operational and includes defined standards for timely, equitable access to quality diagnosis and staging. Time benchmarks (e.g., ≤60 days) adopted and monitored in most jurisdictions. Policy framework establishes affordable access, guaranteeing suitable basic diagnostic packages in the public and private sectors, promoting targeted support for vulnerable groups. Functional referral pathways in most jurisdictions. Standardized protocols in place for evaluation, imaging, biopsy, and HR/HER2 testing, with active quality assurance systems in some jurisdictions. Between 50% and 75% of patients receive complete diagnosis and appropriate staging within two months from initial presentation due to a suspicious finding. **Country characteristics:** Most jurisdictions have functioning diagnostic and staging services. Financial coverage and standardized processes for tissue sampling, pathology, and HR/HER2 testing in place, although precision diagnostics (i.e., germline genetic testing) may not be widely available or publicly covered. Some delays persist in rural or underserved areas.	Comprehensive national policy fully implemented and monitored, ensuring universal access to timely, affordable, and high-quality diagnostic and staging services nationwide. Timeliness indicators embedded in healthcare system dashboards, with operational systematic monitoring. Policy framework mandating full coverage or reimbursement for all diagnostic components both in the public and private sectors, including imaging, pathology, biomarkers, and germline genetic testing when indicated. Universal access ensured through patient navigation services, referral coordination, and equity subsidies. Routine use of quality assurance audits. At least 75% (ideally 90% or more) of patients receive complete diagnosis and appropriate staging within two months of initial presentation due to a suspicious finding. **Country characteristics:** Timely diagnostic services across all jurisdictions. Full financial coverage of all the necessary diagnostic and staging procedures. Continuous quality monitoring. High compliance with statutory timelines.

**Table 6:** Typical policy progress profiles in Dimension C: comprehensive breast cancer management.

**I. Low level of achievement**	**II. Modest level of achievement**
No national policy or framework to guarantee equitable, affordable, and comprehensive multidisciplinary breast cancer care. No standard or monitoring for initiating treatment within three months of first presentation due to a suspicious finding. Treatments for Triple-Negative Breast Cancer (TNBC), HER2+, or HR+ patients often not delivered in accordance with international guidelines. No guarantees for, limited access to, or lack of coverage of supportive services and palliative care (e.g., pain management, physiotherapy, supportive medications, lymphedema management, psycho-oncology, and oncofertility). Lack of consideration for the patients’ perspective or collection of patient-reported outcomes and experience measures (PROMs and PREMs). No national policy or framework to guarantee access to survivorship plans nor follow-up systems post-treatment. <50% of patients complete recommended treatment. **Country characteristics:** Inexistent policy framework or policies lacking necessary elements to focus on multidisciplinary, comprehensive, and patient-centric care. No focus on ensuring provision, coverage, and monitoring of evidence-based, tumor-targeted treatments. Treatment availability depends on isolated tertiary centers, civil society organizations, or the private sector. Frequent treatment abandonment due to cost, access barriers, and lack of follow-up. No focus or provisions in place to ensure availability and access to suitable supportive, palliative, and survivorship care.	A policy or framework outlining the essential requirements for comprehensive, multidisciplinary breast cancer care is under development or exists, but it is not yet fully enforced. Some excellence centers consistently initiate treatment within three months of first presentation due to a suspicious finding. Limited and inconsistent evidence-based access to HER2 therapies, Endocrine Therapy, and chemotherapy for TNBC; availability concentrated in excellence centers. Sporadic provision and coverage of supportive services (such as pain management, physiotherapy, supportive medications, lymphedema management, psycho-oncology and oncofertility) and palliative care, mainly restricted to specialized excellence centers. No systematic use of PROMs and PREMs, but preliminary discussions or pilot projects may exist to incorporate patients’ perspectives. A national policy framework for survivorship care exists, but access is limited to isolated examples within specialized centers of excellence. 50–70% of patients complete recommended treatment. **Country characteristics:** There are efforts to implement comprehensive breast cancer management, but they remain fragmented, with substantial geographic inequities and high financial burden for patients.
**III. Moderate level of achievement**	**IV. Outstanding level of achievement**
National policy operational, defining standards to ensure that comprehensive multidisciplinary breast cancer care is provided in a timely, equitable, and affordable manner, from treatment initiation to completion. Most patients begin treatment within three months of first presentation due to a suspicious finding. HER2 therapies, endocrine therapy, and chemotherapy for TNBC are mostly available in alignment with the guidelines, but coverage gaps persist, despite explicit policy mandates. Systematic supportive and palliative care implemented in some jurisdictions, but not yet fully comprehensive or universal with significant gaps particularly in access and availability of some supportive services. PROMs and PREMs used only in select pilot programs; not yet incorporated nationwide. Structured survivorship plans available in major cancer centers, but not systemwide. 70–85% of patients complete recommended treatment. **Country characteristics:** Referral pathways, access to targeted therapy, and multidisciplinary teams functional in most jurisdictions. Significant progress, but rural or socially vulnerable women face access challenges.	Fully implemented national policy ensures equitable and timely comprehensive multidisciplinary breast cancer care. ≥90% of patients start treatment within 3 months of first presentation due to a suspicious finding. All subtypes (TNBC, HER2+, HR+) receive guideline-adherent therapy consistently. Universal access to palliative and supportive services, including pain management, psychosocial support, physiotherapy, and lymphedema care. PROMs and PREMs routinely collected and used for quality improvement. Nationwide personalized survivorship care plans integrated into standard care. >85% of patients complete recommended treatment. **Country characteristics:** Universal access to guideline-adherent therapy across all breast cancer subtypes, ensuring consistent quality for all patients. Continuous quality assurance and robust referral systems ensure minimal delays or abandonment. Strong integration across the care continuum from treatment initiation to survivorship.

**Table 7:** Typical policy progress profiles in Dimension D: strong and resilient healthcare systems.

**I. Low level of achievement**	**II. Modest level of achievement**
No sustainable funding sources identified for breast cancer services and programs. Severe shortage of specialized healthcare professionals (oncologists, radiologists, pathologists, nurses, etc.). No specialized hospital units or departments in excellence centers or facilities for multidisciplinary breast cancer care. No functional national or regional population-based cancer registry for breast cancer. No systematic reporting of either breast cancer mortality or 5-year survival rates. No national framework for adoption of evidence-based clinical guidelines. No care coordination or patient navigation systems to ensure continuity of care. No platforms for patient engagement in decision-making or service design. **Country characteristics:** Underfunded, limited-capacity healthcare systems. Highly fragmented services, large urban-rural disparities, and absence of system-level monitoring.	Sustainable funding for breast cancer programs proposed or partially allocated. Sporadic recruitment and training initiatives take place at unplanned intervals, but critical shortages of specialized professionals (oncologists, radiologists, pathologists, nurses, etc.) remain. Availability of specialized hospital units or departments for multidisciplinary breast cancer care in excellence centers only. Population-based cancer registry initiated in some jurisdictions, but data collection is not yet comprehensive nationwide. Mortality tracking occurs in select regions at inconsistent intervals; 5-year survival reporting absent. Evidence-based clinical guidelines drafted but no framework to monitor and ensure implementation and adherence nationwide. Pilot care coordination or patient navigation programs operational in a few excellence centers. Occasional consultations with patient groups; no institutionalized mechanisms for patient engagement in decision-making or service design. **Country characteristics:** Basic infrastructure emerging, but service delivery remains uneven, and resources are constrained. Service integration is developing, but healthcare system has limited capacity and lacks patient-centeredness.
**III. Moderate level of achievement**	**IV. Outstanding level of achievement**
Sustainable funding streams established, but periodic shortfalls occur, or overall amount is not adequate. Most required specialists available in urban areas; rural shortages persist. Specialized hospital units or departments for multidisciplinary breast cancer care established in major reference centers, though unevenly distributed. Functional national, population-based registry covers majority of the population with collection of comprehensive data. Annual mortality reporting system in place nationwide; survival estimates available for some regions or at inconsistent intervals. Evidence-based clinical guidelines implemented in most facilities; compliance monitored sporadically or not made publicly available. Structured care coordination or patient navigation systems cover patients treated at all excellence centers, but they are not systematically offered nationally. Patient advisory committees operating in some regions; feedback informs limited-service improvements. **Country characteristics:** Established but not guaranteed funding generates capacity constraints. System functioning in core urban areas, but disparities and implementation gaps remain.	Fully institutionalized funding ensures long-term sustainability for breast cancer programs. Adequate numbers of all specialized professionals; workforce equitably distributed. Well distributed nationwide network of accredited hospital units or departments for multidisciplinary breast cancer care. National population-based cancer registry includes comprehensive and updated data. Annual reporting of both breast cancer mortality and 5-year survival rates integrated into planning. Full implementation, monitoring, and reporting of adherence to evidence-based clinical practice guidelines. Care coordination or patient navigation institutionalized across all levels of care, ensuring continuity. Formal patient engagement platforms integrated into governance and quality improvement. **Country characteristics:** Sustainable funding and healthcare system capacity well-established. Strong, resilient healthcare system ensures equity, quality, and accountability across the entire breast cancer care pathway.

**Table 8:** Definition of key stakeholders.

**Policymakers**
Stakeholders in a country’s legislative and executive branches, including agencies and individuals with formal authority to develop, enact, enforce, and oversee laws, regulations, and national policies on health, including breast cancer. This category covers ministries of health, national cancer control offices, parliamentarians, regulatory authorities, and other government decision-makers at national or subnational levels.
**Multisectoral Stakeholders**
Non-governmental actors at national or subnational levels whose expertise, influence, and resources contribute to the design, implementation, and uptake of breast cancer strategies. This group includes scientific and medical societies, civil society and patient advocacy groups, trade associations, academia and research institutions, think tanks, professional networks, health providers, and philanthropic organizations. They are directly impacted by breast cancer policies but operate outside formal policymaking structures and supranational governance bodies.
**International Community**
Supranational and intergovernmental organizations, their agencies, and funds that influence or coordinate health policy globally or regionally. This includes, among others, the United Nations system, the WHO, the World Bank, the Organisation for Economic Co-operation and Development, and similar bodies, as well as their specialized agencies, technical programs, and financing mechanisms.

**Table 9:** Recommendations for Dimension A: early breast cancer detection.

**I. Low level of achievement**	**II. Modest level of achievement**
**Develop a definition of women at elevated risk of breast cancer** Develop an evidence-based definition of women at elevated risk of breast cancer to delineate the target population for the national early detection strategy and prioritize higher-frequency screening for this group. **Develop risk-assessment tools** With the support of national or international stakeholders, develop context-specific tools for individual risk-assessment to be administered to women during visits to a general practitioner. **Build awareness and educate frontline healthcare workers** Organize a dedicated taskforce to develop and implement a national breast cancer awareness and education strategy, including awareness campaigns targeting women and frontline healthcare workers addressing common misconceptions leading to misdiagnosis or delayed diagnosis, including breast cancer signs and symptoms, risk factors, and family history. **Establish referral pathways** Pilot basic referral pathways for suspicious findings in high-population areas as a model for future scale-up to reach rural or marginalized jurisdictions. **Secure financing** Identify and leverage financing opportunities from international financing facilities (e.g., World Bank, Inter-American Development Bank- IDB, European Bank for Reconstruction and Development- EBRD, Asian Development Bank- ADB, African Development Bank- AdDB) or philanthropic donors to address early detection needs. **Build local technical expertise** Leverage opportunities to receive technical assistance by peer countries, international organizations such as the WHO, consulting agencies, or specialized women’s health advocacy groups.	**Assess progress and gaps** Assess progress in the implementation of the national policy or framework, including guidance for early detection of breast cancer, and identify critical resource gaps to be addressed. **Promote implementation of breast cancer policy** Advocate for stepwise institutionalization of measures from the proposed or recently adopted breast cancer detection plan. **Scale implementation** Identify key jurisdictions to co-pilot a full implementation of the measures laid out in the national policy or framework, including guidance for early detection of breast cancer, and assess adaptations and resources needed for full roll out. **Develop a definition of women at elevated risk of breast cancer** Develop an evidence-based definition of women at elevated risk of breast cancer to delineate the target population for the national early detection strategy and prioritize higher-frequency screening for this group. **Standardize risk-assessment in primary healthcare** Develop context-specific tools for individual risk-assessment to be administered to women during visits to a general practitioner and establish national breast cancer risk-assessment protocols to be rolled out to general practitioners at the national level. **Expand public and workforce awareness** Strengthen health promotion efforts, such as early detection awareness campaigns for the public, and scale education programs for healthcare workers by integrating breast cancer modules into existing national training platforms.
**III. Moderate level of achievement**	**IV. Outstanding level of achievement**
**Integrate early detection in primary health or community care** Ensure systematic integration of breast cancer early detection services across all primary health or community care facilities. **Standardize clinical exams and referral pathways** Ensure uniform implementation of breast exams and referral protocols nationwide. **Establish national monitoring systems** Strengthen national monitoring systems to track public awareness, provider competence, and stage at diagnosis. **Develop a definition of women at elevated risk of breast cancer** Develop an evidence-based definition of women at elevated risk of breast cancer to delineate the target population for the national early detection strategy and prioritize higher-frequency screening for this group. **Define national criteria for screening women at elevated risk** Develop formal criteria to roll out screening for women at elevated risk, based on the specific context and situation (countries may utilize more comprehensive risk assessment approaches when feasible). **Mandate coverage for elevated-risk screening** Expand screening for women at elevated risk, mandating coverage under private insurance and public health services of necessary procedures and interventions. **Embed indicators in universal health care (UHC) and SDG dashboards** Facilitate inclusion of early breast cancer detection metrics in UHC and SDG dashboards, including stage distribution and service access. **Convene multisectoral working groups to streamline referrals** Establish multisectoral regional or local working groups to identify and address barriers to timely referral in rural and peri-urban areas. **Advance operational and implementation research** Support academic research and operational studies to evaluate effectiveness and equity of awareness campaigns and early detection interventions.	**Institutionalize quality assurance for early detection** Implement national quality assurance frameworks for early detection services, including provider certification, audit cycles, and refresher training. **Link indicators to cancer registries** Incorporate stage-at-diagnosis and timeliness indicators into national cancer registries and link them to subnational performance reviews. **Mandate reimbursement of elevated-risk screening, including for family members** Make it mandatory to reimburse screening for women at elevated risk, including BRCA and genetic testing where clinically appropriate. **Incorporate patient-reported measures to strengthen programs** Use patient-reported outcomes and experience measures (PROMs and PREMs) to improve education and navigation services at the primary and community care level, follow-up systems, and awareness campaigns. **Position national institutions as regional leaders** Promote regional leadership in knowledge transfer, supporting peer countries with adaptable policy models, technical assistance, training packages, and evaluation tools. **Co-design awareness strategies with patient advocates** Ensure public awareness strategies are co-designed with patient advocates and academic institutions, so that they reach populations with historically lower early-stage detection. **Institutionalize breast cancer key performance indicators into performance frameworks** Embed breast cancer early detection key performance indicators, such as elevated risk population screening frequency and stage distribution, into facility, healthcare system, and country performance frameworks.

**Table 10:** Recommendations for Dimension B: timely breast cancer diagnosis.

**I. Low level of achievement**	**II. Modest level of achievement**
**Develop a national policy to improve breast cancer diagnosis** Convene a taskforce to draft a national policy to implement improvements for breast cancer diagnosis, outlining objectives, timelines, and actions aligned with the BCCQI, including infrastructure development, workforce training, and referral pathway establishment. **Map diagnostic capacity** Map existing diagnostic capacity (clinical, imaging, pathology, biomarker testing) to identify service gaps and priority jurisdictions for service expansion. **Define diagnostic standards and workflows** Engage academic and clinical institutions to define minimum diagnostic standards and appropriate workflows. **Leverage international assistance** Explore available international technical assistance to design costed implementation plans aligned with existing resource-stratified recommendations. **Produce public education materials** Develop and disseminate public education materials that explain the diagnostic pathway, time sensitivity, and patient rights. **Leverage international best practices** Seek assistance to identify best practices in diagnostic delivery models and possible international experiences to replicate in the country.	**Accelerate implementation of the national framework for breast cancer diagnosis and staging** Begin or consolidate implementation of the national policy or framework with clear timelines (e.g., ≤60 days from presentation) and accountability mechanisms for diagnostic service delivery. **Identify infrastructure gaps** Identify gaps in diagnostic infrastructure (imaging and laboratory) at secondary and regional levels. **Secure adequate resources to strengthen essential infrastructure and pathology services** Identify and leverage financing opportunities from international financing facilities (e.g., World Bank, IDB, EBRD, ADB, AfDB), philanthropic donors, and national resources to enhance essential infrastructure for timely breast cancer diagnosis and expand access to appropriate pathology services, especially biopsy and HR/HER2 testing. **Standardize national referral protocols** Define and disseminate national referral protocols ensuring that timeframes from presentation to diagnosis and staging completion are standardized and monitored. **Define service standards for diagnosis** Establish eligibility and service standards for key diagnostic components, including pathology, HR/HER2 testing, and imaging. **Produce public education materials** Develop and disseminate public education materials that explain the diagnostic pathway, time sensitivity, and patient rights. **Train general and specialized healthcare professionals** Launch training programs for general and specialized healthcare professionals on breast cancer diagnostic protocols and communication of results. **Engage international technical support** Explore available support from the international community for the creation of a network of academic and technical institutions to conduct operational research and data collection on diagnostic timelines and completion rates, drawing on the WHO Collaborating Centers model.
**III. Moderate level of achievement**	**IV. Outstanding level of achievement**
**Ensure appropriate implementation of the national policy or framework for breast cancer diagnosis and staging** Assess and monitor the quality of the diagnostic services provided through the tracking of the standards for timely (e.g., ≤60 days from presentation), equitable access to quality diagnosis and staging. **Institutionalize clinical audits** Institutionalize clinical audit systems in facilities providing diagnostic services to monitor delays and identify causes. **Establish quality assurance frameworks** Establish quality assurance systems for pathology and imaging, considering national participation in international external review programs, such as United Kingdom National External Quality Assessment Service or establishing one nationally with academic or professional organizations. **Produce public education materials** Develop and disseminate public education materials that explain the diagnostic pathway, time sensitivity, and patient rights. **Ensure full financial protection for patients** Ensure financial protection for patients undergoing diagnosis, including coverage of all required tests. **Involve civil society and patient groups** Engage civil society and patient organizations to monitor access to diagnostic services and barriers in vulnerable populations. **Strengthen digital referral systems** Expand digital referral and results systems to reduce turnaround time and promote early identification of dropouts.	**Integrate key diagnostic and staging performance indicators in national cancer registry** Monitor time for completion of diagnosis and staging and record it as supplementary data to the cancer registry. **Guarantee sustained availability of diagnostic services** Ensure sustained universal access to the complete diagnostic and staging process (including clinical evaluation, imaging, tissue sampling, pathological analysis, HR/HER2 testing, and germline genetic testing when indicated) through public financing and/or regulatory enforcement. **Ensure consistency of high-quality, timely diagnostic services** Implement certification mechanisms to standardize and maintain high-quality services that deliver definitive results within the time benchmark for clinical breast evaluation, imaging, biopsy, pathology, HR/HER2, and germline genetic testing when indicated and available. **Adopt real-time tracking systems** Support the establishment of a national dashboard for real-time tracking of all patients referred due to a suspicious finding. **Lead international quality assurance efforts** Promote a cross-border technical exchange program to disseminate best practices and assist countries at lower levels of achievement with knowledge and experience, sharing tools, training, and implementation models to ensure quality in breast cancer diagnosis.

**Table 11:** Recommendations for Dimension C: comprehensive breast cancer management.

**I. Low level of achievement**	**II. Modest level of achievement**
**Develop a national policy for breast cancer management** Initiate the development of a national policy or framework that defines minimum service standards for multidisciplinary breast cancer care from treatment initiation to completion. **Assess service availability** Conduct a service availability and readiness assessment to identify gaps in multidisciplinary, comprehensive and patient-centric care. **Engage experts and civil society** Engage clinical societies and civil society actors to define and prioritize context-adapted models of tumor boards for multidisciplinary breast cancer care, based on well-established and successful examples from similar settings. **Seek international assistance** Collaborate with international actors to access technical and financial assistance for capacity building. **Pilot supportive care programs** Leverage the above-average capabilities of centers of excellence to launch pilot programs for supportive services—such as psycho-oncology and pain management—in tertiary care centers and use these pilots to generate evidence on their impact on patient outcomes, quality of life, and treatment adherence. **Track treatment adherence and abandonment** Develop user-friendly, interim data collection systems to be used at the national level to track treatment adherence and abandonment in selected public hospitals. **Incorporate patient-reported outcomes and experience measures (PROMs and PREMs)** Design context-appropriate mechanisms to begin piloting PROMs and PREMs data collection for disease monitoring, for example, using paper forms or SMS follow-up models. **Raise public awareness on treatment completion** Ensure that tailored information materials and key messages are available in both primary and specialized care settings to educate patients throughout their journey on the importance of treatment adherence and completion, as well as on available support options to address access-related challenges.	**Make the national breast cancer care policy operational** Operationalize the national breast cancer care policy or framework, ensuring accountabilities are well-defined and promoting systematic evaluation and reporting of implementation at the subnational and facility level. **Standardize monitoring timelines** Initiate standard collection of treatment timelines through public registries, whenever possible, to monitor treatment initiation timeliness and attrition rate, based on loss of follow up. **Scale training on treatment protocols for healthcare professionals** Expand training opportunities for healthcare professionals on international treatment protocols by subtype (triple-negative, HER2+, HR+/HER2-), leveraging partnership possibilities and distant learning opportunities. **Ensure equitable access to essential breast cancer drugs** Define benefit packages or drug formularies that ensure access to the different types of treatment options necessary for different cancer subtypes (e.g., chemotherapies, monoclonal antibodies, endocrine therapies, etc.). **Scale-up patient-reported outcomes and experience measures (PROMs and PREMs) data collection** Build up on discussions or expand pilot projects to incorporate patients’ perspectives, through context-appropriate PROMs and PREMs data collection systems. **Develop practical tools to support the implementation of supportive care and basic survivorship care plans** Provide institutions with resources, templates, and guidance to expand supportive services and design tailored survivorship care plans, drawing on the WHO and other international best practices. **Address potential treatment dropouts** Ensure the routine use and analysis of basic patient questionnaires at subnational and facility levels to identify and promptly address potential treatment dropouts.
**III. Moderate level of achievement**	**IV. Outstanding level of achievement**
**Establish treatment quality benchmarks** Enforce monitoring and reporting of breast cancer treatment timeliness and completion benchmarks. **Implement systematic quality-assurance** Establish national quality monitoring and evaluation mechanisms (e.g., clinical audit, systematic reporting) for multidisciplinary, comprehensive, and patient-centric breast cancer care, based on the full set of indicators provided in the BCCQI framework. **Standardize comprehensive supportive and palliative care for breast cancer** Ensure nationwide availability of supportive services across all cancer centers, with minimum requirements for psycho-oncology, physiotherapy, onco-fertility, and symptom and pain management. **Strengthen survivorship programs** Co-develop survivorship programs that include long-term follow-up, education, and access to support groups. **Incentivize systematic monitoring of patient outcomes and experience measures (PROMs and PREMs)** Establish a national working group for the development of easy to implement and nationally standardized tools for systematic monitoring and reporting of PROMs and PREMs and establish incentives for their use at the healthcare facility level. **Promote integration of registries, pharmacy and treatment data** Working along with partners, identify and test sustainable ways to expand data integration across multiple sources (e.g., registries, pharmacy, and treatment centers) to allow for treatment history and adherence tracking through existing cancer registries.	**Ensure systematic reporting of performance based on the breast cancer quality assurance framework** Ensure systematic and transparent reporting of breast cancer system performance, in alignment with the full set of indicators provided in the BCCQI framework. **Guarantee universal access to comprehensive supportive and palliative care for breast cancer** Ensure and monitor availability and access free-of-cost to comprehensive supportive services, including pain management, palliative care, psycho-oncology, physiotherapy, oncofertility, and symptom management. **Embed survivorship care plans** Standardize delivery of survivorship care plans across the country, ensuring provision of the full range of survivor services, including early detection of relapses and family testing. **Ensure comprehensive data recording** Integrate comprehensive information regarding patient characteristics, treatment and disease into cancer registries. **Embed patient outcomes and experience measures (PROMs and PREMs)** Expand use and analysis of patient-reported outcome systems, integrating PROMs and PREMs into health economics and outcomes research and routine service evaluation. **Evaluate long-term impact of breast cancer treatment** Conduct impact assessments of comprehensive breast cancer care on long-term survival and quality of life, using integrated clinical and patient-level data. **Lead cross-country knowledge transfer** Support cross-country capacity building, offering policy tools, training packages, and support for the establishment of context-sensitive national breast cancer quality assurance frameworks.

**Table 12:** Recommendations for Dimension D: strong and resilient healthcare systems.

**I. Low level of achievement**	**II. Modest level of achievement**
**Develop multisectoral financing strategies** Working with the multisectoral stakeholders and international development and financial organizations, develop a resource mobilization plan for breast cancer care programs and services based on sustainable financing sources. **Map the national workforce and service capacity** Establish a national task force to map gaps in breast cancer healthcare workforce, service delivery, and data collection systems, prioritizing underserved populations. **Conduct a baseline infrastructure assessment** Conduct a baseline inventory of diagnostic equipment and specialized healthcare infrastructure for breast cancer to inform policy and resource planning. **Pilot breast cancer-specific data-collection tools** Collaborate with academic institutions and cancer registries to pilot breast cancer-specific data collection tools, including comprehensive information regarding patient characteristics and disease, like, for example, staging at diagnosis and basic sociodemographic indicators. **Develop a guideline adoption roadmap** Develop a roadmap for national adoption and phased implementation of international clinical guidelines tailored to local resource levels, with emphasis on extended training for healthcare professionals. **Consult stakeholders on care coordination or patient navigation** Begin stakeholder consultations to define minimum requirements and feasibility for care coordination or patient navigation models and patient engagement platforms.	**Institutionalize and embed breast cancer financing mechanisms** Strengthen national policies to establish dedicated financing for breast cancer detection, treatment, survivorship, and data systems, embedding them through legal and budgetary instruments. **Scale up workforce training** Expand investment in training programs to increase the availability of breast cancer-specialized healthcare professionals, prioritizing rural and underserved regions. **Strengthen providers’ compliance with quality standards** Develop national quality standards for breast cancer units and facilities, drawing on international frameworks and guidelines, and reinforce compliance through targeted incentives to ensure adherence to minimum standards. **Expand coverage and improve consistency of data collected through population-based cancer registries** Establish a dedicated government task force to work with population-based cancer registries to develop and enforce national protocols for cancer registration, including standardized collection of stage-at-diagnosis data and linkage with mortality and survival data. **Disseminate evidence-based clinical guidelines** Promote the adaptation and dissemination of evidence-based breast cancer clinical practice guidelines and strengthen their uptake through continuous training and targeted monitoring mechanisms. **Improve coordination or navigation across the continuum of care** Conduct pilot programs for structured care coordination models or patient navigation in public hospitals, including training of navigators and documentation protocols. **Establish patient advisory committees** Institutionalize a national patient advisory committee for breast cancer, ensuring regular feedback on service design and system-level reforms and transparently report on its impact on actual policies.
**III. Moderate level of achievement**	**IV. Outstanding level of achievement**
**Expand annual budget lines dedicated to breast cancer** Ensure that annual budget lines dedicated to breast cancer in national and subnational health budgets are sufficient to meet needs, and work with multisectoral stakeholders to mobilize additional resources, where required, to cover both service delivery and the integration of innovations. **Ensure consistent planning for breast cancer workforce** Integrate breast cancer workforce planning into broader national human resource for health strategies, using service-to-population ratios to project needs. **Strengthen compliance with national quality standards** Establish a nationwide mandatory facility accreditation system for breast cancer-specialized hospital units and departments, tying resource allocation to compliance with multidisciplinary infrastructure and service standards. **Ensure full population-wide breast cancer registries integrating multi-source information** Promote systematic collection and multi-source integration of core patient and disease data—ensuring full coverage of demographics and disease stage at diagnosis—while progressively expanding registries to include survival and mortality outcomes. **Embed clinical practice guideline compliance monitoring** Integrate monitoring of compliance with clinical practice guidelines into national healthcare frameworks, using structured clinical audits or regular facility-level reporting to ensure that findings feed directly into quality improvement cycles. **Define care coordination or patient navigation approach for national adoption** Conduct a national stakeholder-based assessment across care levels to identify the most suitable care coordination or patient navigation approach to establish at the country level and develop a national framework for breast cancer care coordination or cancer patient navigation to be implemented. **Embed patient participation in decision-making** Establish formal patient engagement channels through legal mandate for participation in national and subnational breast cancer planning and quality review committees.	**Institutionalize multiyear financing frameworks** Ensure inclusion of multiyear investment frameworks for breast cancer within national health strategies and UHC roadmaps, guaranteeing full coverage of multidisciplinary, patient-centric, and comprehensive breast cancer care. **Benchmark and monitor workforce distribution** Develop a suitable tool for workforce planning, need forecasting, benchmarking workforce-to-need ratios at subnational level, like, for example, a dynamic dashboard to monitor staffing gaps and distribution of breast cancer specialists. **Ensure transparency of breast cancer-specialized hospital unit and department performance** Develop a publicly available dashboard or a systematic report series to disseminate findings of the yearly survey conducted to grant accreditation through the mandatory facility accreditation system. **Ensure comprehensiveness of population-based breast cancer registries** Systematically record breast cancer stage at diagnosis and relapses and other relevant disease-related characteristics during long-term follow-up, ensuring sufficient and reliable data to make the outcome indicators of mortality or 5-year survival available. **Link breast cancer data to broader country performance assessments** Link population-wide breast cancer data to healthcare system performance dashboards and SDG/UHC monitoring frameworks for continuous policy feedback. **Establish a legal responsibility for malpractices linked to non-compliance with Clinical Practice Guidelines (CPGs)** Establish legal accountability or performance-based resource allocation systems that reward compliance with CPGs to strengthen patient protection. **Formalize care coordination and patient navigation programs** Allocate funds and resources to ensure continued sustainability and expansion of national care coordination or patient navigation programs, encouraging and incentivizing multi-stakeholder engagement and oversight. **Institutionalize patient-informed breast cancer service performance assessment** Expand the terms of reference of the formal patient-engagement channels to the continuous collection and analysis of patient outcomes and experience measures (PREMs and PROMs), establishing annual or biannual reporting on patient experience (for example, through support for formal and systemic engagement of academic institutions to report periodically on breast cancer services performance from the patient point of view).

## Data Availability

Not applicable.

## Supplementary Materials

The following supporting information can be downloaded at https://www.mdpi.com/article/10.3390/ijerph23020207/s1, Supplementary Material S1: 100 Country Assessment; Tables S1–S6: Table S1. Indicators used for the 100 Country Assessment by BCCQI Dimension [50,51,52,53]; Table S2. Country Scoring Criteria Applied for the 100 Country Assessment by BCCQI Dimension; Table S3. Country Progress Ranking for the Americas Region by Total Score [54,55,56,57,58,59,60,61,62,63,64,65,66,67,68,69,70,71]; Table S4. Country Progress Ranking for the Asia Pacific Region by Total Score [72,73,74,75,76,77,78,79,80,81,82,83,84,85,86,87,88]; Table S5. Country Progress Ranking for the European Region by Total Score [89,90,91,92,93,94,95,96,97,98,99,100,101,102,103,104,105,106,107,108,109,110,111,112,113,114,115,116,117,118,119,120,121,122,123]; Table S6. Country Progress Ranking for the Middle East, Turkey, and Africa (META) Region by Total Score [124,125,126,127,128,129,130,131,132,133,134,135,136,137,138,139,140,141,142,143,144,145,146,147,148,149,150,151,152,153]; Supplementary Material S2; Tables S7–S23: Table S7. Examples of the stakeholder types considered within each of the three main categories of stakeholders responsible for the BCCQI implementation; Table S8. Stakeholders involved in recommendations for Dimension A on early breast cancer detection for countries with low level of achievement; Table S9. Stakeholders involved in recommendations for Dimension A on early breast cancer detection for countries with modest level of achievement; Table S10. Stakeholders involved in recommendations for Dimension A on early breast cancer detection for countries with moderate level of achievement; Table S11. Stakeholders involved in recommendations for Dimension A on early breast cancer detection for countries with outstanding level of achievement; Table S12. Stakeholders involved in recommendations for Dimension B on timely breast cancer diagnosis for countries with low level of achievement; Table S13. Stakeholders involved in recommendations for Dimension B on timely breast cancer diagnosis for countries with modest level of achievement; Table S14. Stakeholders involved in recommendations for Dimension B on timely breast cancer diagnosis for countries with moderate level of achievement; Table S15. Stakeholders involved in recommendations for Dimension B on timely breast cancer diagnosis for countries with outstanding level of achievement; Table S16. Stakeholders involved in recommendations for Dimension C on comprehensive breast cancer management for countries with low level of achievement; Table S17. Stakeholders involved in recommendations for Dimension C on comprehensive breast cancer management for countries with modest level of achievement; Table S18. Stakeholders involved in recommendations for Dimension C on comprehensive breast cancer management for countries with moderate level of achievement; Table S19. Stakeholders involved in recommendations for Dimension C on comprehensive breast cancer management for countries with outstanding level of achievement; Table S20. Stakeholders involved in recommendations for Dimension D on strong and resilient healthcare systems for countries with low level of achievement; Table S21. Stakeholders involved in recommendations for Dimension D on strong and resilient healthcare systems for countries with modest level of achievement; Table S22. Stakeholders involved in recommendations for Dimension D on strong and resilient healthcare systems for countries with moderate level of achievement; Table S23. Stakeholders involved in recommendations for Dimension D on strong and resilient healthcare systems for countries with outstanding level of achievement.
