# Supplementary material for: Improving Breast Cancer Outcomes Through Quality Care: Call to Action for the Implementation of the Breast Cancer Care Quality Index (BCCQI)
Source: Int J Environ Res Public Health. 2026 Feb 6;23(2):207. doi: 10.3390/ijerph23020207 (PMC12940849; doi:10.3390/ijerph23020207)
Supplement: Supplementary file 1 [file ijerph-23-00207-s001.zip › ijerph-4087785-supplementary.pdf]

Supplementary Material

Table of contents

SUPPLEMENTARY MATERIAL S1 .... **3**

100 Country Assessment 3

Table S1. Indicators used for the 100 Country Assessment by BCCQI Dimension 3

Table S2. Country Scoring Criteria Applied for the 100 Country Assessment by BCCQI Dimension .5

Table S3. Country Progress Ranking for the Americas Region by Total Score 6

Table S4. Country Progress Ranking for the Asia Pacific Region by Total Score 7

Table S5. Country Progress Ranking for the European Region by Total Score 8

Table S6. Country Progress Ranking for the Middle East, Turkey, and Africa (META) Region by Total Score 9

SUPPLEMENTARY MATERIAL S2 .... **11**

Definition of Key Stakeholders 11

Table S7. Examples of the stakeholder types considered within each of the three main categories of stakeholders responsible for the BCCQI implementation. 11

A. Early Breast Cancer Detection 14

Table S8. Stakeholders involved in recommendations for Dimension A on early breast cancer detection for countries with low level of achievement 14

Table S9. Stakeholders involved in recommendations for Dimension A on early breast cancer detection for countries with modest level of achievement 15

Table S10. Stakeholders involved in recommendations for Dimension A on early breast cancer detection for countries with moderate level of achievement 16

Table S11. Stakeholders involved in recommendations for Dimension A on early breast cancer detection for countries with outstanding level of achievement 17

B. Timely Breast Cancer Diagnosis 19

Table S12. Stakeholders involved in recommendations for Dimension B on timely breast cancer diagnosis for countries with low level of achievement 19

Table S13. Stakeholders involved in recommendations for Dimension B on timely breast cancer diagnosis for countries with modest level of achievement 20

Table S14. Stakeholders involved in recommendations for Dimension B on timely breast cancer diagnosis for countries with moderate level of achievement 21

Table S15. Stakeholders involved in recommendations for Dimension B on timely breast cancer diagnosis for countries with outstanding level of achievement 22

C. Comprehensive Breast Cancer Management 24

Table S16. Stakeholders involved in recommendations for Dimension C on comprehensive breast cancer management for countries with low level of achievement 24
Table S17. Stakeholders involved in recommendations for Dimension C on comprehensive breast cancer management for countries with modest level of achievement 25
Table S18. Stakeholders involved in recommendations for Dimension C on comprehensive breast cancer management for countries with moderate level of achievement 26
Table S19. Stakeholders involved in recommendations for Dimension C on comprehensive breast cancer management for countries with outstanding level of achievement 27
D. STRONG AND RESILIENT HEALTHCARE SYSTEMS 29
Table S20. Stakeholders involved in recommendations for Dimension D on strong and resilient healthcare systems for countries with low level of achievement 29
Table S21. Stakeholders involved in recommendations for Dimension D on strong and resilient healthcare systems for countries with modest level of achievement 30
Table S22. Stakeholders involved in recommendations for Dimension D on strong and resilient healthcare systems for countries with moderate level of achievement 31
Table S23. Stakeholders involved in recommendations for Dimension D on strong and resilient healthcare systems for countries with outstanding level of achievement 32

Supplementary Material S1

100 Country Assessment

This Supplementary Material presents an exploratory application of the Breast Cancer Care Quality Indicators (BCCQI) framework to a cohort of 100 countries, illustrating how country-level assessments using the BCCQI could be operationalized in practice. Countries were selected based on absolute breast cancer mortality, prioritizing those reporting more than 500 annual deaths, while excluding very small countries with high mortality rates that may not be consistently captured in international databases, as well as countries with significant concerns regarding data reliability (e.g., autocratic regimes).

The analysis aimed to evaluate countries' progress across the four dimensions of the BCCQI—(A) Early Detection, (B) Timely Diagnosis, (C) Comprehensive Management, and (D) Strong and Resilient Healthcare Systems—using globally available, standardized data to maximize cross-national comparability. Some practical challenges were encountered in the assessment. Country-level information on breast cancer across the four BCCQI dimensions was sparse, with numerous gaps in key indicators and inconsistencies in reporting. To mitigate these limitations, alternative data points were identified from established global databases, prioritizing sources with broad geographic coverage and standardized metrics.

Consequently, this exercise serves as a structured test of the BCCQI's applicability, demonstrating how a uniform framework can be implemented across diverse national contexts. The analysis also highlights the need for the adoption of a shared self-assessment tool to enable coordinated reporting and analysis for baseline establishment and progress monitoring of the BCCQI, particularly in the absence of comprehensive data. In this sense, while constrained by data availability, the exercise lays the foundation for developing a globally applicable self-reporting questionnaire to track progress and support iterative improvements in breast cancer care as evidence gaps are progressively addressed.

A standardized research template was developed in Microsoft Excel to systematically capture relevant indicators within each BCCQI dimension. Selection of indicators was guided by their availability in internationally recognized data sources, ensuring comparability across countries. Key datasets developed by global organizations—including World Health Organization's (WHO) Cancer Country Profiles, IARC's CanScreen5, World Bank's Tracking Universal Health Coverage – 2023 Global Monitoring Report, and Global Essential Medicines—were leveraged wherever possible to provide a coherent cross-country assessment framework. Table S1 shows the final list of indicators for this analysis.

Table S1. Indicators used for the 100 Country Assessment by BCCQI Dimension

BCCQI Dimension	Indicator	Source
**A. Early breast cancer detection**	A1. Availability of Breast Cancer screening program<br>A2. Availability of mammographs	WHO's Cancer Country Profiles [50]

**B. Timely breast cancer diagnosis**	B1. Availability of pathology services<br>B2. Availability of radiation oncologists<br>B3. Availability of medical & pathology lab scientists<br>B4. Availability of nuclear medicine physicians<br>B5. Availability of CT scanners	WHO's Cancer Country Profiles [50]
**C. Comprehensive breast cancer management**	C1. Treatment services (chemotherapy, surgery, radiotherapy)<br>C2. Available breast cancer medicines<br>C3. Supportive services<br>C4. Palliative care<br>C5. Treatment coverage	1. WHO's Cancer Country Profiles [50]<br>2. Global Essential Medicines [51]<br>3. IARC's CanScreen5 [52]<br>4. WHO's Cancer Country Profiles [50]<br>5. World Bank's Tracking Universal Health Coverage – 2023 Global Monitoring Report [53]
**D. Strong and resilient healthcare systems**	D1. Density of cancer centers<br>D2. Cancer registry<br>D3. Clinical guidelines	WHO's Cancer Country Profiles [50]

A team of five trained researchers conducted data collection, with each assigned to 20 countries. Upon completion of data extraction, scoring and classification criteria were established to develop a four-point scale, ranging from 1 (low) to 4 (outstanding), categorizing performance within each dimension. Table S2 presents the scoring criteria used. Automated Excel formulas were applied to implement the four-point scales to each dimension, and countries were subsequently ranked within their respective regions according to their aggregated scores across all four dimensions. To ensure relevance and meaningfulness of the output, a group of experts was engaged to identify outliers and correct limitations in the dataset. Based on this assessment, additional *ad hoc* research was conducted on a limited number of countries to validate and adjust the scoring as needed. Tables S3 through S6 present the results of this analysis. Countries for which additional research was conducted are marked with *letters in parenthesis*, indicating which dimensions were supplemented with other information sources described in the References section of this Annex. Different levels of progress are shown in the following color code:

- **1 = Low = Red**
- **2 = Modest = Orange**
- **3 = Moderate = Gold**
- **4 = Outstanding = Green**

Table S2. Country Scoring Criteria Applied for the 100 Country Assessment by BCCQI Dimension

**Dimension A.** Early Breast Cancer Detection	
Outstanding	Countries that fulfill criteria regarding **Screening program** AND **Screening equipment availability for high-risk populations**.
Moderate	Countries that fulfill criteria regarding **Screening program** BUT DO NOT FULFILL **Screening equipment availability for high-risk populations**.
Modest	Countries that fulfill criteria regarding **Screening equipment availability for high-risk populations** BUT DO NOT FULFILL **Screening program**.
Low	Countries DO NOT FULFILL criteria for any of these indicators.
**Dimension B.** Timely Breast Cancer Diagnosis	
Outstanding	Countries that fulfill criteria regarding **Availability of services** AND **Human Resources** AND **Equipment**.
Moderate	Countries that fulfill criteria regarding **Availability of services** BUT DO NOT fulfill both **Human Resources** AND **Equipment**.
Modest	Countries that ONLY fulfill criteria regarding **Availability of services**.
Low	Countries that DO NOT fulfill criteria regarding **Availability of services**.
**Dimension C.** Comprehensive Breast Cancer Management	
Outstanding	Countries that fulfill criteria regarding **Treatment** AND **Supportive Services and Palliative Care** AND **UHC Coverage**.
Moderate	Countries that fulfill criteria regarding **Treatment** BUT FULFILL criteria of either or both **Supportive Services and Palliative Care** AND **UHC Coverage**.
Modest	Countries that DO NOT fulfill criteria regarding **Treatment** AND DO NOT fulfill either or both **Supportive Services and Palliative Care** AND **UHC Coverage**.
Low	Countries that DO NOT FULFILL criteria for any of these indicators.
**Dimension D.** Strong and Resilient Healthcare Systems	
Outstanding	Countries that fulfill criteria regarding **Density of Healthcare Services** AND **Cancer Registry** AND **Guidelines**.
Moderate	Countries that fulfill criteria for **Cancer Registry** AND **Guidelines** but DO NOT fulfill criteria for **Density of Healthcare Services**.

Modest	Countries that DO NOT FULFILL both **Cancer Registry** AND **Guidelines** BUT fulfill one or two out of these three indicators.
Low	Countries that DO NOT fulfill any of these indicators.

Table S3. Country Progress Ranking for the Americas Region by Total Score

Country	Region	A. Early Breast Cancer Detection	B. Timely Breast Cancer Diagnosis	C. Compre-hensive Breast Cancer Management	D. Strong and Resilient Health care Systems	Total Score	Overall Progress Level<br><br>Quartile 4: Outstanding<br>Quartile 3: Moderate<br>Quartile 2: Modest<br>Quartile 1: Low	Progress %
USA (D) [54]	AMERICAS	4	4	4	3	15	Moderate	94%
Canada (B,D) [55]	AMERICAS	4	3	4	3	14	Moderate	88%
Chile	AMERICAS	4	4	2	3	13	Moderate	81%
Uruguay (D) [56]	AMERICAS	4	3	2	4	13	Moderate	81%
Colombia (A,B,C,D) [57]	AMERICAS	3	3	3	3	12	Modest	75%
Peru (A) [58]	AMERICAS	3	2	3	3	11	Modest	69%
Argentina	AMERICAS	3	2	2	3	10	Modest	63%
Brazil (A,B,C,D) [59–61]	AMERICAS	3	2	2	3	10	Modest	63%
Dominican Republic (A) [62,63]	AMERICAS	2	3	3	2	10	Modest	63%
Ecuador (D) [64]	AMERICAS	3	2	2	3	10	Modest	63%
Mexico (A,B,C) [65,66]	AMERICAS	3	3	2	2	10	Modest	63%
Bolivia	AMERICAS	2	3	2	2	9	Low	56%
Cuba (A) [67,68]	AMERICAS	2	2	2	2	8	Low	50%
Paraguay	AMERICAS	3	3	1	1	8	Low	50%

Guatemala (A,B) [69]	AMERICAS	2	2	1	2	7	Low	44%
Haiti (A,B,C,D) [70]	AMERICAS	1	1	1	1	4	Low	25%
Venezuela (A,C) [71]	AMERICAS	1	1	1	1	4	Low	25%

Table S4. Country Progress Ranking for the Asia Pacific Region by Total Score

Country	Region	A. Early Breast Cancer Detection	B. Timely Breast Cancer Diagnosis	C. Compre-hensive Breast Cancer Management	D. Strong and Resilient Healthcare Systems	Total Score	Overall Progress Level<br><br>Quartile 4: Outstanding<br>Quartile 3: Moderate<br>Quartile 2: Modest<br>Quartile 1: Low	Progress %
Australia (C,D) [72]	APAC	4	4	4	3	15	Moderate	94%
New Zealand (B,D) [73,74]	APAC	4	4	4	3	15	Moderate	94%
South Korea	APAC	4	3	4	3	14	Moderate	88%
Japan (C,D) [75–77]	APAC	3	4	3	3	13	Moderate	81%
Malaysia (C)[78]	APAC	4	4	2	3	13	Moderate	81%
Thailand (B,C,) [79,80]	APAC	3	2	3	4	12	Modest	75%
China	APAC	3	3	2	3	11	Modest	69%
India (C,D) [81,82]	APAC	3	2	2	3	10	Modest	63%
Kazakhstan	APAC	4	2	2	2	10	Modest	63%
Azerbaijan	APAC	3	3	1	2	9	Low	56%
Indonesia (B,C,D) [83–85]	APAC	3	2	2	2	9	Low	56%
Iran (A,B,D) [86–88]	APAC	2	3	2	2	9	Low	56%
Sri Lanka	APAC	3	3	1	2	9	Low	56%
Viet Nam	APAC	3	1	1	3	8	Low	50%
Bangladesh	APAC	3	1	1	2	7	Low	44%
Philippines	APAC	3	1	1	2	7	Low	44%

Nepal	APAC	3	1	1	1	6	Low	38%
Pakistan	APAC	1	3	1	1	6	Low	38%
Cambodia	APAC	1	1	1	2	5	Low	31%

Table S5. Country Progress Ranking for the European Region by Total Score

Country	Region	A. Early Breast Cancer Detection	B. Timely Breast Cancer Diagnosis	C. Compre-hensive Breast Cancer Management	D. Strong and Resilient Healthcare Systems	Total Score	Overall Progress Level<br><br>Quartile 4: Outstanding<br>Quartile 3: Moderate<br>Quartile 2: Modest<br>Quartile 1: Low	Progress %
Austria (C) [89]	EUROPE	4	4	4	4	16	Outstanding	100%
Belgium (A) [90,91]	EUROPE	4	4	4	4	16	Outstanding	100%
Denmark (A) [92]	EUROPE	4	4	4	4	16	Outstanding	100%
Finland	EUROPE	4	4	4	4	16	Outstanding	100%
Ireland (A,B) [93]	EUROPE	4	4	4	4	16	Outstanding	100%
Lithuania	EUROPE	4	4	4	4	16	Outstanding	100%
Norway (C,D) [94,95]	EUROPE	4	4	4	4	16	Outstanding	100%
Slovakia	EUROPE	4	4	4	4	16	Outstanding	100%
Switzerland (D,E) [96–98]	EUROPE	4	4	4	4	16	Outstanding	100%
France (A,B,D) [99]	EUROPE	3	4	4	4	15	Moderate	94%
Germany (A,C) [100]	EUROPE	3	4	4	4	15	Moderate	94%
Italy (C) [101]	EUROPE	4	4	3	4	15	Moderate	94%
United Kingdom (A,B,C,D) [102–104]	EUROPE	4	4	4	3	15	Moderate	94%
Netherlands (B,C) [105]	EUROPE	4	3	4	3	14	Moderate	88%
Portugal (C) [106]	EUROPE	4	4	2	4	14	Moderate	88%
Spain (B,C,D) [107]	EUROPE	4	4	3	3	14	Moderate	88%
Sweden (A,C,D) [108]	EUROPE	4	4	3	3	14	Moderate	88%
Czech Republic (C)	EUROPE	3	3	4	3	13	Moderate	81%
Georgia	EUROPE	4	3	4	2	13	Moderate	81%
Romania (A,D)	EUROPE	2	3	4	3	12	Modest	75%

Croatia (A,B,C) [109]	EUROPE	3	3	2	3	11	Modest	69%
Serbia (C) [110]	EUROPE	3	3	3	2	11	Modest	69%
Bosnia and Herzegovina (A,B,C,D) [111–113]	EUROPE	2	3	3	2	10	Modest	63%
Greece (A,B,C) [114]	EUROPE	3	2	3	2	10	Modest	63%
Hungary (A,C,D) [115]	EUROPE	2	3	2	3	10	Modest	63%
Russia (A,C) [116]	EUROPE	2	3	3	2	10	Modest	63%
Bulgaria (A,C,D) [117]	EUROPE	2	2	2	3	9	Low	56%
Poland (A,B) [118,119]	EUROPE	3	2	2	2	9	Low	56%
Belarus (A,B,D) [120–122]	EUROPE	1	2	2	3	8	Low	50%
Moldova	EUROPE	3	2	1	2	8	Low	50%
Ukraine (A,D) [123]	EUROPE	2	2	2	2	8	Low	50%

Table S6. Country Progress Ranking for the Middle East, Turkey, and Africa (META) Region by Total Score

Country	Region	A. Early Breast Cancer Detection	B. Timely Breast Cancer Diagnosis	C. Compre-hensive Breast Cancer Management	D. Strong and Resilient Healthcare Systems	Total Score	Overall Progress Level<br><br>Quartile 4: Outstanding<br>Quartile 3: Moderate<br>Quartile 2: Modest<br>Quartile 1: Low	Progress %
Jordan (A,B,D) [124–127]	META	3	3	3	3	12	Modest	75%
Algeria (A,B,D) [128]	META	3	3	2	3	11	Modest	69%
Israel (C) [129–131]	META	4	4	3	4	15	Moderate	94%
Türkiye (A,B,D) [132]	META	3	3	2	3	11	Modest	69%
Kingdom of Saudi Arabia (C) [133–138]	META	4	3	3	4	14	Moderate	88%
Egypt (A,B) [139]	META	3	3	2	2	10	Modest	63%
Lebanon (A,B) [140–142]	META	3	3	1	2	9	Low	56%
Morocco (A,B) [143]	META	3	3	1	2	9	Low	56%

Tunisia (A,B) [144,145]	META	3	3	1	2	9	Low	56%
Kenya (A,E,D) [146–148]	META	2	3	1	2	8	Low	50%
South Africa (A,B,C) [149]	META	3	2	2	3	10	Modest	63%
Guinea (B) [150]	META	3	2	1	2	8	Low	50%
Iraq (A,B) [151]	META	3	2	1	2	8	Low	50%
Mozambique	META	3	2	2	2	9	Low	56%
Uzbekistan	META	3	2	2	2	9	Low	56%
Zambia	META	3	3	1	2	9	Low	56%
Zimbabwe	META	3	2	2	2	9	Low	56%
Burkina Faso	META	3	2	1	2	8	Low	50%
Nigeria (D) [152]	META	3	3	1	1	8	Low	50%
Senegal	META	3	2	1	2	8	Low	50%
Syria (A) [153]	META	1	2	1	1	5	Low	31%
Tanzania	META	3	2	1	2	8	Low	50%
Côte d'Ivoire	META	3	1	1	2	7	Low	44%
Ethiopia	META	3	1	1	2	7	Low	44%
Rwanda	META	3	1	1	2	7	Low	44%
Uganda	META	1	1	1	3	6	Low	38%
Angola	META	1	2	1	1	5	Low	31%
Cameroon	META	1	2	1	1	5	Low	31%
Ghana	META	1	1	1	2	5	Low	31%
Madagascar	META	1	1	1	2	5	Low	31%
Malawi	META	1	1	1	2	5	Low	31%

Supplementary Material S2

Definition of Key Stakeholders

The BCCQI co-authors identified three main categories of stakeholders responsible for promoting and ensuring the implementation of the BCCQI: (1) policymakers, (2) multisectoral stakeholders, and (3) the international community.

For the purpose of this Call to Action, these categories are defined according to the descriptions presented in Table 8.

To further support global, regional, and national stakeholders in implementing this Call to Action, these categories have been disaggregated to specify the key actors encompassed within each of the three stakeholder groups. Table A7 below outlines some examples of the stakeholder types considered within each category for the purposes of this Call to Action.

Table S7. Examples of the stakeholder types considered within each of the three main categories of stakeholders responsible for the BCCQI implementation.

**Policymakers**	• **Government (GOV) -** President/Prime Minister, Council of Ministers<br>• **Health Agencies (HA) -** Ministry of Health, Public Health Agency & National Health Institutes, Centers for Disease Control, National Regulatory & Procurement Agencies<br>• **Health Advisory Boards (HAB) -** National Health Advisory Board, National Supreme Councils, Chief Medical Officers, Medical Ethics Board
**Multisectoral Stakeholders**	• **Scientific/Medical Societies (SMS) –** Healthcare Professional Associations, Medical and Scientific Societies, Public Health Societies<br>• **Patient groups (PAG) -** Patient Advocacy Group, Disease-Specific Patient Organization, Health Rights Network<br>• **Research Centres (RC) –** Academia, Research Institutes, Think Tanks<br>• **Health & Laboratory Providers (HLP) –** Primary Care & Community Health Centers, Hospitals and Hospital Networks, Clinical Care & Diagnostic Services Providers, Outpatient Care Providers

	• **Trade & Pharma Associations (TPA) -** Pharmaceutical Associations, Industry Associations, Health Trade Organizations<br>• **Supranational and intergovernmental health policy and development bodies and agencies (SIB) –** World Health Organization, United Nations (UN), Organisation for Economic Co-operation and Development (OECD), European Union (EU), African Union (AU), Pan American Health Organization (PAHO), Asia-Pacific Economic Cooperation (APEC), Association of Southeast Asian Nations (ASEAN), African Union (AU)<br>• **Funding bodies and financing mechanisms (FBM) –** The World Bank, International Monetary Fund, Gavi, the Vaccine Alliance, Global Fund to Fight AIDS, Tuberculosis and Malaria (GFATM), Multilateral Regional Development Banks, Bilateral Development Programs (e.g., USAID, PEPFAR, Foreign, Commonwealth & Development Office (FCDO), Swedish International Development Cooperation Agency (SIDA), Japan International Cooperation Agency, JICA), UN-Systems Programs& Funds (UNICEF, PAHO Revolving and Strategic Funds, UNDP), Philanthropic Funds (e.g., Bill & Melinda Gates Foundation, Clinton Health Access Initiative)
**International Community**	

The following sections of this Annex provide additional detail and guidance on the respective roles and spheres of influence of each stakeholder type listed above as they relate to the implementation of the recommendations outlined in Section 6 of this document.

Each stakeholder's contribution is defined under one or more of the following roles:

- **Developer** – Responsible for developing guidance, policies, or frameworks outlined in the recommendation.
- **Implementer** – Responsible for enacting policies, executing the key activities or programs described in the recommendation.
- **Technical Expert** – Responsible for consulting or providing technical input, advice, or feedback related to the activities outlined in the recommendation.

- **Advocate –** Responsible for raising awareness, generating momentum, garnering support, and upholding accountability for the acceptance, execution, and implementation of the activities outlined in the recommendation.

A. Early Breast Cancer Detection

Tables S8 to S11 summarize stakeholder roles and responsibilities in implementing the recommendations for Dimension A: early breast cancer detection.

Table S8. Stakeholders involved in recommendations for Dimension A on early breast cancer detection for countries with low level of achievement

I. Recommendations for countries with LOW level of achievement	GOV	HA	HAB	SIB	FBM	SMS	PAG	RC	HLP	TPA
**1. Develop a definition of women at elevated risk of breast cancer**<br>Develop an evidence-based definition of women at elevated risk of breast cancer to delineate the target population for the national early detection strategy and prioritize higher-frequency screening for this group.	-	D I	D T	-	-	T	T A	D T	I	A
**2. Develop risk-assessment tools**<br>With the support of national or international stakeholders develop context-specific tools for individual risk-assessment to be administered to women during visits to a general practitioner.	-	D	-	T	-	D T	-	D T	I	A
**3. Build awareness and educate frontline healthcare workers**<br>Organize a dedicated taskforce to develop and implement a national breast cancer awareness and education strategy, including awareness campaigns targeting women and frontline healthcare workers addressing common misconceptions leading to misdiagnosis or delayed diagnosis, including breast cancer signs and symptoms, risk factors, and family history.	D	I	T	T	-	T	D I<br>T A	T	T	A
**4. Establish referral pathways**<br>Pilot basic referral pathways for suspicious findings in high-population areas as a model for future scale-up to reach rural or marginalized jurisdictions.	-	D	D T	T	-	-	-	I T	I T	-
**5. Secure financing**<br>Identify and leverage financing opportunities from international financing facilities (e.g., World Bank, Inter-American Development Bank- IDB, European Bank for Reconstruction and Development- EBRD, Asian Development Bank- ADB, African Development Bank- AdDB) or philanthropic donors to address early detection needs.	I	T	T	-	D	-	A	-	-	-
**6. Build local technical expertise**<br>Leverage opportunities to receive technical assistance by peer countries, international organizations such as the WHO, consulting agencies, or specialized women's health advocacy groups.	-	D I	T	T	-	I	A	T I	A	T

D=Developers I=Implementers T=Technical Expertise A= Advocacy

Table S9. Stakeholders involved in recommendations for Dimension A on early breast cancer detection for countries with modest level of achievement

II. Recommendations for countries with MODEST level of achievement	GOV	HA	HAB	SIB	FBM	SMS	PAG	RC	HLP	TPA
**1. Assess progress and gaps**<br>Assess progress in the implementation of the national policy or framework including guidance for early detection of breast cancer and identify critical resource gaps to be addressed.	-	D I	T	T	-	T	T A	D I	T	D T
**2. Promote breast cancer policy implementation**<br>Advocate for stepwise institutionalization of measures from the proposed or recently adopted breast cancer detection plan.	-	I	T	T	-	T A	A	A	A	T A
**3. Scale implementation**<br>Identify key jurisdictions to co-pilot a full implementation of the measures laid out in the national policy or framework including guidance for early detection of breast cancer and assess adaptations and resources needed for full roll out.	-	D I	T	T	I	T	A	I T	I	-
**4.Develop a definition of women at elevated risk of breast cancer**<br>Develop an evidence-based definition of women at elevated risk of breast cancer to delineate the target population for the national early detection strategy and prioritize higher-frequency screening for this group.	-	D I	D T	-	-	T	T A	D T	I	A
**5. Standardize risk-assessment in primary healthcare**<br>Develop context-specific tools for individual risk-assessment to be administered to women during visits to a general practitioner and establish national breast cancer risk-assessment protocols to be rolled out to general practitioners at the national level.	-	D	D T	T	-	D T	A	D T	I	T A
**6. Expand public and workforce awareness**<br>Strengthen health promotion efforts, such as early detection awareness campaigns for the public and scale education programs for healthcare workers by integrating breast cancer modules into existing national training platforms.	I	D	T	-	-	I	I	T	I	A

D=Developers I=Implementers T=Technical Expertise A= Advocacy

Table S10. Stakeholders involved in recommendations for Dimension A on early breast cancer detection for countries with moderate level of achievement

III. Recommendations for countries with MODERATE level of achievement	GOV	HA	HAB	SIB	FBM	SMS	PAG	RC	HLP	TPA
**1. Integrate early detection in primary health or community care**<br>Ensure systematic integration of breast cancer early detection services across all primary health or community care facilities.	-	D	T	T	-	T	A	T	I	A
**2. Standardize clinical exams and referral pathways**<br>Ensure uniform implementation of breast exams and referral protocols nationwide.	-	D I	T	T	-	T A	A	T	I	-
**3. Establish national monitoring systems**<br>Strengthen national monitoring systems to track public awareness, provider competence, and stage at diagnosis.	D	I	T	T	-	-	-	T	I	A
**4. Develop a definition of women at elevated risk of breast cancer**<br>Develop an evidence-based definition of women at elevated risk of breast cancer to delineate the target population for the national early detection strategy and prioritize higher-frequency screening for this group.	-	D I	D T	-	-	T	T A	D T	I	A
**5. Define national criteria for screening women at elevated risk**<br>Develop formal criteria to roll out screening for women at elevated risk, based on the specific context and situation (countries may utilize more comprehensive risk assessment approaches when feasible).	-	I	T	-	-	D T	A	D T	I	A
**6. Mandate coverage for elevated-risk screening**<br>Expand screening for women at elevated risk, mandating coverage under private insurance and public health services.	D	I	T	-	-	-	A	-	-	A
**7. Embed indicators in UHC and Sustainable Development Goals (SDG) dashboards**<br>Facilitate inclusion of early breast cancer detection metrics in UHC and SDG dashboards, including stage distribution and service access.	-	I	T	D	-	-	A	T	-	A
**8. Convene multisectoral working groups to streamline referrals**<br>Establish multisectoral regional or local working groups to identify and address barriers to timely referral in rural and peri-urban areas.	-	D	I	-	-	D	D A	D	D	D
**9. Advance operational and implementation research**	-	-	T	-	-	-	-	D I	D	A

Support academic research and operational studies to evaluate effectiveness and equity of awareness campaigns and early detection interventions.										

D=Developers I=Implementers T=Technical Expertise A= Advocacy

Table S11. Stakeholders involved in recommendations for Dimension A on early breast cancer detection for countries with outstanding level of achievement

IV. Recommendations for countries with OUTSTANDING level of achievement	GOV	HA	HAB	SIB	FBM	SMS	PAG	RC	HLP	TPA
**1. Institutionalize quality assurance for early detection** Implement national quality assurance frameworks for early detection services, including provider certification, audit cycles, and refresher training.	-	I	T	-	-	T	A	-	I	A
**2. Link indicators to cancer registries** Incorporate stage-at-diagnosis and timeliness indicators into national cancer registries and link them to subnational performance reviews.	-	I	T	-	-	D	A	T	I	A
**3. Mandate reimbursement of elevated-risk screening, including for family members** Make it mandatory to reimburse screening for women at elevated risk, including BRCA testing where clinically appropriate.	I	D	T	-	-	-	A	-	-	A
**4. Incorporate patient-reported measures to strengthen programs** Use patient-reported experience and outcome measures (PREMs and PROMs) to improve education and navigation services at the primary and community care level, follow-up systems, and awareness campaigns.	D	I	T	T	-	-	D A	T	-	-
**5. Position national institutions as regional leaders** Promote regional leadership in knowledge transfer, supporting peer countries with adaptable policy models, technical assistance, training packages, and evaluation tools.	D	D I	-	D	-	-	A	-	-	-
**6. Co-design awareness strategies with patient advocates**	-	D	T	-	-	D	D I T A	-	-	A

Ensure public awareness strategies are co-designed with patient advocates and academic institutions, so that they reach populations with historically lower early-stage detection.										
**7. Institutionalize breast cancer key performance indicators into performance frameworks**<br>Embed early breast cancer detection key performance indicators, such as elevated risk population screening frequency and stage distribution, into facility, healthcare system, and country performance frameworks.	-	D I	D	T	-	-	-	T	I	-

D=Developers I=Implementers T=Technical Expertise A= Advocacy

B. Timely Breast Cancer Diagnosis

Tables S12 to S15 summarize stakeholder roles and responsibilities in implementing the recommendations for Dimension B: timely breast cancer diagnosis.

Table S12. Stakeholders involved in recommendations for Dimension B on timely breast cancer diagnosis for countries with low level of achievement

I. Recommendations for countries with LOW level of achievement	GOV	HA	HAB	SIB	FBM	SMS	PAG	RC	HLP	TPA
**1. Develop a national policy to improve breast cancer diagnosis** Convene a taskforce to draft a national policy to implement improvements for breast cancer diagnosis, outlining objectives, timelines, and actions aligned with the BCCQI, including infrastructure development, workforce training, and referral pathway establishment.	D	I	T	-	-	-	A	T	I	A
**2. Map diagnostic capacity** Map existing diagnostic capacity (clinical, imaging, pathology, biomarker testing) to identify service gaps and priority jurisdictions for service expansion.	-	I	T	-	-	-	-	D	D	-
**3. Define diagnostic standards and workflows** Engage academic and clinical institutions to define minimum diagnostic standards and appropriate workflows.	-	D I	T	-	-	T	A	T	D	-
**4. Leverage international assistance** Explore available international technical assistance to design costed implementation plans aligned with existing resource-stratified recommendations.	D	I	-	D	D	-	-	-	-	-
**5. Produce public education materials** Develop and disseminate public education materials that explain the diagnostic pathway, time sensitivity, and patient rights.	-	A	-	-	-	-	D I T A	T	-	A
**6. Leverage international best practices** Seek assistance to identify best practices in diagnostic delivery models and possible international experiences to replicate in the country.	-	I	-	D	-	T	A	T	I	D

D=Developers I=Implementers T=Technical Expertise A= Advocacy

Table S13. Stakeholders involved in recommendations for Dimension B on timely breast cancer diagnosis for countries with modest level of achievement

II. Recommendations for countries with MODEST level of achievement	GOV	HA	HAB	SIB	FBM	SMS	PAG	RC	HLP	TPA
**1. Accelerate implementation of the national framework for breast cancer diagnosis and staging**<br>Begin or consolidate implementation of the national policy or framework with clear timelines (e.g., ≤60 days from presentation) and accountability mechanisms for diagnostic service delivery.	D	I	T	-	-	T	A	T	-	-
**2. Identify infrastructure gaps**<br>Identify gaps in diagnostic infrastructure (imaging and laboratory) at secondary and regional levels.	-	I	T	-	-	-	-	D	-	-
**3. Secure adequate resources to strengthen essential infrastructure and pathology services**<br>Identify and leverage financing opportunities from international financing facilities (e.g., World Bank, IDB, EBRD, ADB, AfDB), philanthropic donors, and national resources to enhance essential infrastructure for timely breast cancer diagnosis and expand access to appropriate pathology services, especially biopsy and HR/HER2 testing.	D	I	T	-	D	T	-	T	A	-
**4. Standardize national referral protocols**<br>Define and disseminate national referral protocols ensuring that timeframes from presentation to diagnosis and staging completion are standardized and monitored.	D	D	T	-	-	D	-	T	I	-
**5. Define service standards for diagnosis**<br>Establish eligibility and service standards for key diagnostic components, including pathology, HR/HER2 testing, and imaging.	D	D I	T	-	-	D	-	T	D	-
**6. Produce public education materials**<br>Develop and disseminate public education materials that explain the diagnostic pathway, time sensitivity, and patient rights.	-	D	-	-	-	-	D I T A	T	-	A
**7. Train general and specialized healthcare professionals**	-	I	T	T	-	D	-	T	I	A

Launch training programs for general and specialized healthcare professionals on breast cancer diagnostic protocols and communication of results.										
**8. Engage international technical support**<br>Explore available support from the international community for the creation of a network of academic and technical institutions to conduct operational research and data collection on diagnostic timelines and completion rates, drawing on the WHO Collaborating Centers model.	-	I	T	D		-	-	-	I	-

D=Developers I=Implementers T=Technical Expertise A= Advocacy

Table S14. Stakeholders involved in recommendations for Dimension B on timely breast cancer diagnosis for countries with moderate level of achievement

III. Recommendations for countries with MODERATE level of achievement	GOV	HA	HAB	SIB	FBM	SMS	PAG	RC	HLP	TPA
**1. Ensure appropriate implementation of the national policy or framework for breast cancer diagnosis and staging**<br>Assess and monitor the quality of the diagnostic services provided through the tracking of the standards for timely (e.g., ≤60 days from presentation), equitable access to quality diagnosis and staging.<br>Scale implementation of national diagnostic protocols, including time-tracking mechanisms across facilities.	D	I	T	T	-	T	A	-	I	-
**2. Institutionalize clinical audits**<br>Institutionalize clinical audit systems in facilities providing diagnostic services to monitor delays and identify causes.	-	I	T	-	-	T	-	-	D	-
**3. Establish quality assurance frameworks**<br>Establish quality assurance systems for pathology and imaging, considering national participation in international external review programs, such as United Kingdom National External Quality Assessment Service or establishing one nationally with academic or professional organizations.	-	I	D	-	D	T	-	-	D	-
**4. Produce public education materials**<br>Develop and disseminate public education materials that explain the diagnostic pathway, time sensitivity, and patient rights.	-	D T	T	-	-	D T I	D I T A	T I	I	D T A

**5. Ensure full financial protection for patients**<br>Ensure financial protection for patients undergoing diagnosis, including coverage of all required tests.	D	I	T	-	-	-	A	-	-	-
**6. Involve civil society and patient groups**<br>Engage civil society and patient organizations to monitor access to diagnostic services and barriers in vulnerable populations.	-	D	D	-	-	-	D I	D	T	A
**7. Strengthen digital referral systems**<br>Expand digital referral and results systems to reduce turnaround time and promote early identification of drop-outs.	-	D I	T	-	-	-	-	T	I	-

D=Developers I=Implementers T=Technical Expertise A= Advocacy

Table S15. Stakeholders involved in recommendations for Dimension B on timely breast cancer diagnosis for countries with outstanding level of achievement

IV. Recommendations for countries with OUTSTANDING level of achievement	GOV	HA	HAB	SIB	FBM	SMS	PAG	RC	HLP	TPA
**1. Develop a definition of women at elevated risk of breast cancer**<br>Develop an evidence-based definition of women at elevated risk of breast cancer to delineate the target population for the national early detection strategy and prioritize higher-frequency screening for this group.	-	D I	D T	-	-	T	T A	D T	I	A
**2. Develop risk-assessment tools**<br>With the support of national or international stakeholders develop context-specific tools for individual risk-assessment to be administered to women during visits to a general practitioner.	-	D	-	T	-	D T	-	D T	I	A
**3. Build awareness and educate frontline healthcare workers**<br>Organize a dedicated taskforce to develop and implement a national breast cancer awareness and education strategy, including awareness campaigns targeting women and frontline healthcare workers addressing common misconceptions leading to misdiagnosis or delayed diagnosis, including breast cancer signs and symptoms, risk factors, and family history.	D	I	T	T	-	T	D I<br>T A	T	T	A
**4. Establish referral pathways**	-	D	D T	T	-	-	-	I T	I T	-

Pilot basic referral pathways for suspicious findings in high-population areas as a model for future scale-up to reach rural or marginalized jurisdictions.										
**5. Secure financing**<br>Identify and leverage financing opportunities from international financing facilities (e.g., World Bank, IDB, EBRD, ADB, AfDB) or philanthropic donors to address early detection needs.	I	T	T	-	D	-	A	-	-	-

D=Developers I=Implementers T=Technical Expertise A= Advocacy

C. Comprehensive Breast Cancer Management

Tables S16 to S19 summarize stakeholder roles and responsibilities in implementing the recommendations for Dimension C: comprehensive breast cancer management.

Table S16. Stakeholders involved in recommendations for Dimension C on comprehensive breast cancer management for countries with low level of achievement

I. Recommendations for countries with LOW level of achievement	GOV	HA	HAB	SIB	FBM	SMS	PAG	RC	HLP	TPA
**1. Develop a national policy for breast cancer management** Initiate the development of a national policy or framework that defines minimum service standards for multidisciplinary breast cancer care from treatment initiation to completion.	D	D I	T	T	-	T	A	T	T	-
**2. Assess service availability** Conduct a service availability and readiness assessment to identify gaps in multidisciplinary, comprehensive and patient-centric care.	-	I	I T	-	-	I T	I T A	T	D	T A
**3. Engage experts and civil society** Engage clinical societies and civil society actors to define and prioritize context-adapted models of tumor boards for multidisciplinary breast cancer care, based on well-established and successful examples of in similar settings.	-	I	D	T	-	D	D A	D T	D	A
**4. Seek international assistance** Collaborate with international actors to access technical and financial assistance for capacity building.	-	I	T	D	D	T	A	T	T	A
**5. Pilot supportive care programs** Leverage the above-average capabilities of centers of excellence to launch pilot programs for supportive services—such as psycho-oncology and pain management—in tertiary care centers and use these pilots to generate evidence on their impact on patient outcomes, quality of life, and treatment adherence.	-	I	T	T	-	D	A	T	D	-
**6. Track treatment adherence and abandonment** Develop user-friendly, interim data collection systems to be used at the national level to track treatment adherence and abandonment in selected public hospitals.	-	D	T	T	I	D T	A	D T	D T	A
**7. Incorporate patient-reported outcome or experience measures (PROMs, PREMs)**		D A	D I T A			I T A	A	I T A		

Design context-appropriate mechanisms to begin piloting PROMs and PREMs data collection for disease monitoring, for example, using paper forms or SMS follow-up models.										
**8. Raise public awareness on treatment completion**<br>Ensure that tailored information materials and key messages are available in both primary and specialized care settings to educate patients throughout their journey on the importance of treatment adherence and completion, as well as on available support options to address access-related challenges.	-	D I	T	I	I	D I A	D I A	T	I T	-

D=Developers I=Implementers T=Technical Expertise A= Advocacy

Table S17. Stakeholders involved in recommendations for Dimension C on comprehensive breast cancer management for countries with modest level of achievement

II. Recommendations for countries with MODEST level of achievement	GOV	HA	HAB	SIB	FBM	SMS	PAG	RC	HLP	TPA
**1. Make the national cancer care policy operational**<br>Operationalize the national cancer care policy or framework, ensuring accountabilities are well-defined and promoting systematic evaluation and reporting of implementation at the subnational and facility level.	D	I	T	T	-	-	A	-	D	-
**2. Standardize monitoring timelines**<br>Initiate standard collection of treatment timelines through public registries, whenever possible, to monitor treatment initiation timeliness and attrition rate, based on loss of follow up.	D	I	T	-	-	D	A	-	I	-
**3. Scale HCP training on treatment protocols**<br>Expand training opportunities for healthcare professionals on international treatment protocols by subtype (triple-negative, HER2+, HR+/HER2-), leveraging partnership possibilities and distant learning opportunities.	-	I	T	T	-	D	A	T	I	A
**4. Ensure equitable access to essential breast cancer drugs**<br>Define benefit packages or drug formularies that ensure access to the different types of treatment options necessary for different cancer subtypes (e.g., chemotherapies, monoclonal antibodies, endocrine therapies, etc.).	I	D	T	-	-	T	A	T	I	A
**5. Scale-up PROM/PREM data collection**		D A	D I T A			I T A	A	I T A		

Build on ongoing discussions or scale pilot projects to better incorporate patients' perspectives using context-appropriate PROMs and PREMs data-collection systems.										
**6. Develop practical tools to support the implementation of supportive care and basic survivorship care plans**<br>Provide institutions with resources, templates, and guidance to expand supportive services and design tailored survivorship care plans, drawing on WHO and other international best practice.	-	D	T	-	-	I	D A	T	D	A
**7. Address potential treatment dropouts**<br>Ensure the routine use and analysis of basic patient questionnaires at subnational and facility levels to identify and promptly address potential treatment dropouts.	-	D	T	-	-	-	D A	T	I	A

D=Developers I=Implementers T=Technical Expertise A= Advocacy

Table S18. Stakeholders involved in recommendations for Dimension C on comprehensive breast cancer management for countries with moderate level of achievement

III. Recommendations for countries with MODERATE level of achievement	GOV	HA	HAB	SIB	FBM	SMS	PAG	RC	HLP	TPA
**1. Establish treatment quality benchmarks**<br>Enforce monitoring and reporting of breast cancer treatment timeliness and completion benchmarks.	I	D I	T	T	T	D I A	A	I T A	I	A
**2. Implement systematic quality-assurance**<br>Establish national quality monitoring and evaluation mechanisms (e.g., clinical audit, systematic reporting) for multidisciplinary, comprehensive, and patient-centric breast cancer care, based on the full set of indicators provided in the BCCQI framework.	-	I	T	-	-	T	-	-	D	-
**3. Standardize comprehensive supportive and palliative care for breast cancer**<br>Ensure nationwide availability of supportive services across all cancer centers, with minimum requirements for psycho-oncology, physiotherapy, onco-fertility, and symptom management, and pain management.	D	I	T	-	-	T	A	-	D	-
**4. Strengthen survivorship programs**	-	D	T	-	-	T A	D I A	T A	D	A

Co-develop survivorship programs that include long-term follow-up, education, and access to support groups.										
**5. Incentivize systematic monitoring of patient experience**<br>Establish a national working group for the development of easy to implement and nationally standardized tools for systematic monitoring and reporting of PROMs and PREMs and establish incentives for their use at the healthcare facility level.	D	D I	T	T	T	T A	T A	T A	D A	-
**6. Promote integration of registries, pharmacy and treatment data**<br>Working along with partners, identify and test sustainable ways to expand data integration across multiple sources (e.g., registries, pharmacy, and treatment centers) to allow treatment history and adherence tracking through existing cancer registries.	-	D	T	-	-	T	-	-	I	-

D=Developers I=Implementers T=Technical Expertise A= Advocacy

Table S19. Stakeholders involved in recommendations for Dimension C on comprehensive breast cancer management for countries with outstanding level of achievement

IV. Recommendations for countries with OUTSTANDING level of achievement	GOV	HA	HAB	SIB	FBM	SMS	PAG	RC	HLP	TPA
**1. Ensure systematic reporting of performance based on the breast cancer quality assurance framework**<br>Ensure systematic and transparent reporting of breast cancer system performance, in alignment with the full set of indicators provided in the BCCQI framework.	-	D I	T	T	-	I T A	A	I T	I T	A
**2. Guarantee universal access to comprehensive supportive and palliative care for breast cancer**<br>Ensure and monitor availability and access free-of-cost to comprehensive supportive services, including pain management, palliative care, psycho-oncology, physiotherapy, oncofertility, and symptom management.	D	I	T	T	-	T	I T A	I T	I	T A
**3. Embed survivorship care plans**<br>Standardize delivery of survivorship care plans across the country, ensuring provision of the full range of survivor services, including early detection of relapses and family testing.	-	D I	T	T	I	I T	A	T A	D I	A
**4. Ensure comprehensive data recording**	-	I	T	T	-	T A	A	T A	D I	A

Integrate comprehensive information regarding patient characteristics, treatment and disease into cancer registries.										
**5. Embed PROMs and PREMs**<br>Expand use and analysis of patient-reported outcome systems, integrating PROMs and PREMs into health economics and outcomes research (HEOR) and routine service evaluation.	-	I	D	T	-	T	D	-	I	-
**6. Evaluate long-term impact of breast cancer treatment**<br>Conduct impact assessments of comprehensive care on long-term survival and quality of life, using integrated clinical and patient-level data.	-	D	T	T	I	I T A	T A	D I	D	T
**7. Lead cross-country knowledge transfer**<br>Support cross-country capacity building, offering policy tools, training packages, and support for the establishment of context-sensitive national breast cancer quality assurance frameworks.	-	D	T	I	-	T	-	-	D	T

D=Developers I=Implementers T=Technical Expertise A= Advocacy

D. Strong and Resilient Healthcare Systems

Tables S20 to S23 summarize stakeholder roles and responsibilities in implementing the recommendations for Dimension D: strong and resilient healthcare systems.

Table S20. Stakeholders involved in recommendations for Dimension D on strong and resilient healthcare systems for countries with low level of achievement

I. Recommendations for countries with LOW level of achievement	GOV	HA	HAB	SIB	FBM	SMS	PAG	RC	HLP	TPA
**1. Develop multisectoral financing strategies**<br>Working with the multisectoral stakeholders and international development and financial organizations, develop a resource mobilization plan for breast cancer care programs and services based on sustainable financing sources.	I	D	T	-	T	A	A	T A	D A	T A
**2. Map the national workforce and service capacity**<br>Establish a national task force to map gaps in breast cancer healthcare workforce, service delivery, and data collection systems, prioritizing underserved populations.	-	I	T	-	-	T	-	I	D	-
**3. Conduct a baseline infrastructure assessment**<br>Conduct a baseline inventory of diagnostic equipment and specialized healthcare infrastructure for breast cancer to inform policy and resource planning.	-	I	T	-	-	-	-	I	D	-
**4. Pilot breast cancer-specific data-collection tools**<br>Collaborate with academic institutions and cancer registries to pilot breast cancer-specific data collection tools, including comprehensive information regarding patient characteristics and disease, like, for example, staging at diagnosis and basic sociodemographic indicators.	-	D I	T	T	T	D I T	A	D I T	D I T	T A
**5. Develop a guideline adoption roadmap**<br>Develop a roadmap for national adoption and phased implementation of international clinical guidelines tailored to local resource levels, with emphasis on extended training for healthcare professionals.	-	I	T	T	-	D	-	T	D	-
**6. Consult stakeholders on care coordination or patient navigation**	-	D	I	-	-	-	D I A	-	D	-

Begin stakeholder consultations to define minimum requirements and feasibility for care coordination or patient navigation models and patient engagement platforms.										

D=Developers I=Implementers T=Technical Expertise A= Advocacy

Table S21. Stakeholders involved in recommendations for Dimension D on strong and resilient healthcare systems for countries with modest level of achievement

II. Recommendations for countries with MODEST level of achievement	GOV	HA	HAB	SIB	FBM	SMS	PAG	RC	HLP	TPA
**1. Institutionalize and embed breast cancer financing mechanisms**<br>Strengthen national policies to establish dedicated financing for breast cancer detection, treatment, survivorship, and data systems, embedding them through legal and budgetary instruments.	I	D	-	-	T	A	A	T	A	A
**2. Scale up workforce training**<br>Expand investment in training programs to increase the availability of breast cancer-specialized healthcare professionals, prioritizing rural and underserved regions.	-	I	T	T	T	A	A	T	D A	A
**3. Strengthen providers' compliance with quality standards**<br>Develop national quality standards for breast cancer units and facilities, drawing on international frameworks and guidelines, and reinforce compliance through targeted incentives to ensure adherence to minimum standards.	A	I	T	T	-	D A	A	T D	D	A
**4. Expand coverage and improve consistency of data collected through population-based cancer registries**<br>Establish a dedicated government task force to work with population-based cancer registries to develop and enforce national protocols for cancer registration, including standardized collection of stage-at-diagnosis data and linkage with mortality and survival data.	-	I	T	T	-	D	-	D	I	-
**5. Disseminate evidence-based clinical guidelines**<br>Promote the adaptation and dissemination of evidence-based breast cancer clinical practice guidelines and strengthen their uptake through continuous training and targeted monitoring mechanisms.	-	I	T	-	-	D T A	A	I T	D T A	T A
**6. Improve coordination or navigation across the continuum of care**<br>Conduct pilot programs for structured care coordination models or patient navigation in public hospitals, including training of navigators and documentation protocols.	-	I	T	-	-	-	D I A	-	D	-

**7. Establish patient advisory committees**<br>Institutionalize a national patient advisory committee for breast cancer, ensuring regular feedback on service design and system-level reforms and transparently report on its impact on actual policies.	-	D	D	-	-	-	D I A	-	D	-

D=Developers I=Implementers T=Technical Expertise A= Advocacy

Table S22. Stakeholders involved in recommendations for Dimension D on strong and resilient healthcare systems for countries with moderate level of achievement

III. Recommendations for countries with MODERATE level of achievement	GOV	HA	HAB	SIB	FBM	SMS	PAG	RC	HLP	TPA
**1. Expand annual budget lines dedicated to breast cancer**<br>Ensure that annual budget lines dedicated to breast cancer in national and subnational health budgets are sufficient to meet needs, and work with multisectoral stakeholders to mobilize additional resources, where required, to cover both service delivery and the integration of innovations.	I	D	-	-	-	-	-	-	-	-
**2. Ensure consistent planning for breast cancer workforce**<br>Integrate breast cancer workforce planning into broader national human resource for health strategies, using service-to-population ratios to project needs.	D I	D T	T	T	-	D T A	A	D T A	D	A
**3. Strengthen compliance with national quality standards**<br>Establish a nationwide mandatory facility accreditation system for breast caner-specialized hospital units and departments, tying resource allocation to compliance with multidisciplinary infrastructure and service standards.	-	I	T	T	-	D	-	-	D	-
**4. Ensure full population-wide breast cancer registries integrating multi-source information**<br>Promote systematic collection and multi-source integration of core patient and disease data—ensuring full coverage of demographics and disease stage at diagnosis—while progressively expanding registries to include survival and mortality outcomes.	-	D I	T	T	-	D I T	A	D I T	D I	A
**5. Embed clinical practice guideline compliance monitoring**<br>Integrate monitoring of compliance with clinical practice guidelines into national healthcare frameworks, using structured clinical audits or regular facility-level reporting to ensure that findings feed directly into quality improvement cycles.	-	I	T	-	-	D	-	-	D	-
**6. Formalize care coordination or patient navigation approach for national adoption**<br>Conduct a national stakeholder-based assessment across care levels to identify the most suitable care coordination or patient navigation approach to establish at the country level	-	I	T	-	-	-	D I A	-	D	-

and develop a national framework for breast cancer care coordination or cancer patient navigation to be implemented.										
**7. Embed patient participation in decision-making**<br>Establish formal patient engagement channels through legal mandate for participation in national and subnational breast cancer planning and quality review committees.	-	I	D	-	-	-	D I<br>A	-	D	-

D=Developers I=Implementers T=Technical Expertise A= Advocacy

Table S23. Stakeholders involved in recommendations for Dimension D on strong and resilient healthcare systems for countries with outstanding level of achievement

IV. Recommendations for countries with OUTSTANDING level of achievement	GOV	HA	HAB	SIB	FBM	SMS	PAG	RC	HLP	TPA
**1. Institutionalize multiyear financing frameworks**<br>Ensure inclusion of multiyear investment frameworks for breast cancer within national health strategies and UHC roadmaps, guaranteeing full coverage of multidisciplinary, patient-centric, and comprehensive breast cancer care.	I	D	-	-	-	-	-	-	-	-
**2. Benchmark and monitor workforce distribution**<br>Develop a suitable tool for workforce planning, need forecasting, benchmarking workforce-to-need ratios at subnational level, like, for example, a dynamic dashboard to monitor staffing gaps and distribution of breast cancer specialists	-	I	T	T	-	D	-	T	D	-
**3. Ensure transparency of breast cancer-specialized hospital unit and department performance**<br>Develop a publicly available dashboard or a systematic report series to disseminate findings of the yearly survey conducted to grant accreditation through the mandatory facility accreditation system.	-	I	T	T	T	T	A	-	D	A
**4. Ensure comprehensiveness of population-based breast cancer registries**<br>Systematically record breast cancer stage at diagnosis and relapses and other relevant disease-related characteristics during long-term follow-up, ensuring sufficient and reliable data to make the outcome indicators of mortality or 5-year survival available	-	I	T	T	-	D	-	D	I	-
**5. Link breast cancer data to broader country performance assessments**<br>Link population-wide breast cancer data to health system performance dashboards and SDG/UHC monitoring frameworks for continuous policy feedback.	-	D I	T	-	-	I	A	I	I	A

**6. Establish a legal responsibility for malpractices linked to non-compliance with Clinical Practice Guidelines (CPGs)**<br>Establish legal accountability or performance-based resource allocation systems that reward compliance with CPGs to strengthen patient protection	I	D	T	-	-	D	A	T	D	-
**7. Formalize care coordination and patient navigation programs**<br>Allocate funds and resources to ensure continued sustainability and expansion of national care coordination or patient navigation programs, encouraging and incentivizing multi-stakeholder engagement and oversight.	-	I	T	-	-	-	D I A	-	D	-
**8. Institutionalize patient-informed breast cancer service performance assessment**<br>Expand the terms of reference of the formal patient-engagement channels to the continuous collection and analysis of PREMs and PROMs, establishing annual or biannual reporting on patient experience (for example, through support for formal and systemic engagement of academic institutions to report periodically on breast cancer services performance from the patient point of view).	-	I	D	-	-	-	D I A	-	D	-

D=Developers I=Implementers T=Technical Expertise A= Advocacy
